# Identification of *C21orf59* and *ATG2A* as novel determinants of renal function-related traits in Japanese by exome-wide association studies

**DOI:** 10.18632/oncotarget.16696

**Published:** 2017-03-30

**Authors:** Yoshiji Yamada, Jun Sakuma, Ichiro Takeuchi, Yoshiki Yasukochi, Kimihiko Kato, Mitsutoshi Oguri, Tetsuo Fujimaki, Hideki Horibe, Masaaki Muramatsu, Motoji Sawabe, Yoshinori Fujiwara, Yu Taniguchi, Shuichi Obuchi, Hisashi Kawai, Shoji Shinkai, Seijiro Mori, Tomio Arai, Masashi Tanaka

**Affiliations:** ^1^ Department of Human Functional Genomics, Advanced Science Research Promotion Center, Mie University, Tsu, Japan; ^2^ CREST, Japan Science and Technology Agency, Kawaguchi, Japan; ^3^ Computer Science Department, College of Information Science, University of Tsukuba, Tsukuba, Japan; ^4^ RIKEN Center for Advanced Intelligence Project, Tokyo, Japan; ^5^ Department of Computer Science, Nagoya Institute of Technology, Nagoya, Japan; ^6^ Department of Internal Medicine, Meitoh Hospital, Nagoya, Japan; ^7^ Department of Cardiology, Kasugai Municipal Hospital, Kasugai, Japan; ^8^ Department of Cardiovascular Medicine, Inabe General Hospital, Inabe, Japan; ^9^ Department of Cardiovascular Medicine, Gifu Prefectural Tajimi Hospital, Tajimi, Japan; ^10^ Department of Molecular Epidemiology, Medical Research Institute, Tokyo Medical and Dental University, Tokyo, Japan; ^11^ Section of Molecular Pathology, Graduate School of Health Care Sciences, Tokyo Medical and Dental University, Tokyo, Japan; ^12^ Research Team for Social Participation and Community Health, Tokyo Metropolitan Institute of Gerontology, Tokyo, Japan; ^13^ Research Team for Promoting Support System for Home Care, Tokyo Metropolitan Institute of Gerontology, Tokyo, Japan; ^14^ Research Team for Social Participation and Health Promotion, Tokyo Metropolitan Institute of Gerontology, Tokyo, Japan; ^15^ Center for Promotion of Clinical Investigation, Tokyo Metropolitan Geriatric Hospital, Tokyo, Japan; ^16^ Department of Pathology, Tokyo Metropolitan Geriatric Hospital, Tokyo, Japan; ^17^ Department of Clinical Laboratory, Tokyo Metropolitan Geriatric Hospital, Tokyo, Japan

**Keywords:** chronic kidney disease, hyperuricemia, glomerular filtration rate, serum uric acid, exome-wide association study

## Abstract

We have performed exome-wide association studies to identify genetic variants that influence renal function-related traits or confer susceptibility to chronic kidney disease or hyperuricemia in Japanese. Exome-wide association studies for estimated glomerular filtration rate and the serum concentration of creatinine were performed with 12,565 individuals, that for the serum concentration of uric acid with 9934 individuals, and those for chronic kidney disease or hyperuricemia with 5161 individuals (3270 cases, 1891 controls) or 11,686 individuals (2045 cases, 9641 controls), respectively. The relation of genotypes of single nucleotide polymorphisms to estimated glomerular filtration rate or the serum concentrations of creatinine or uric acid was examined by linear regression analysis, and that of allele frequencies of single nucleotide polymorphisms to chronic kidney disease or hyperuricemia was examined with Fisher's exact test. The exome-wide association studies revealed that 25, seven, and six single nucleotide polymorphisms were significantly (*P* <1.21 × 10^−6^) associated with estimated glomerular filtration rate or the serum concentrations of creatinine or uric acid, respectively, and that 49 and 35 polymorphisms were significantly associated with chronic kidney disease or hyperuricemia, respectively. Subsequent multivariable logistic regression analysis with adjustment for covariates revealed that four and three single nucleotide polymorphisms were related (*P* < 0.05) to chronic kidney disease or hyperuricemia, respectively. Among polymorphisms identified in the present study, rs76974938 [C/T (D67N)] of *C21orf59* and rs188780113 [G/A (R478C)] of *ATG2A* may be novel determinants of estimated glomerular filtration rate and chronic kidney disease or of the serum concentration of uric acid, respectively.

## INTRODUCTION

Chronic kidney disease (CKD) is an important risk factor for cardiovascular disease as well as end-stage renal disease [1–6]. Genome-wide association studies (GWASs) have identified various genes and loci for renal function-related traits in European ancestry [7–11], African-American [12, 13], or East Asian [14] populations. A recent meta-analysis of GWASs in European ancestry populations identified 53 loci that were significantly related to estimated glomerular filtration rate (eGFR) [15].

Hyperuricemia is a crucial risk factor for gout, a common inflammatory type of arthritis [16, 17], cardiovascular disease [18], and cancer [19]. Although multiple renal transporters contribute to the maintenance of normal circulating uric acid levels by mediating the excretion or reabsorption of uric acid in the proximal kidney tubules, the underlying mechanisms of such homeostasis have not been fully elucidated [20]. The heritability of the serum concentration of uric acid has been estimated to be 40% [21], suggesting that genetic variants contribute to regulation of this parameter by influencing uric acid synthesis, excretion, or reabsorption [21, 22]. Previous GWASs have identified single nucleotide polymorphisms (SNPs) significantly associated with the serum uric acid concentration or the prevalence of gout [23–29]. A large-scale GWAS in European ancestry populations identified 28 loci that influence the serum concentration of uric acid [30].

Most genetic variants identified in these various previous GWASs were common SNPs with a minor allele frequency (MAF) of >5% and a small individual effect size. Given that these common SNPs may explain a small fraction of the heritability of CKD or hyperuricemia, it is expected that low-frequency (0.5% ≤ MAF < 5%) or rare (MAF <0.5%) variants with larger effect sizes also contribute to the genetic architecture of these conditions [31]. Although several polymorphisms have been found to be significantly associated with renal function [14] or gout [32, 33] in Japanese, genetic variants—including low-frequency and rare variants—that influence renal function-related traits or contribute to predisposition to CKD or hyperuricemia in the Japanese population have not been identified definitively.

We have now performed exome-wide association studies (EWASs) with the use of exome array-based genotyping methods in order to identify SNPs—especially low-frequency or rare coding variants with moderate to large effect sizes—that influence renal function-related traits or confer susceptibility to CKD or hyperuricemia in Japanese. Given that most of the known low-frequency or rare variants were not included in arrays adopted in previous related GWASs, we used Illumina human exome arrays that provide coverage of functional SNPs in entire exons including low-frequency and rare variants.

## RESULTS

### EWASs for eGFR and serum concentrations of creatinine and uric acid

We examined the relation of genotypes for 41,352 SNPs that passed quality control to eGFR or the serum concentration of creatinine in 12,565 subjects by linear regression analysis. Manhattan plots of the EWASs for eGFR and the serum creatinine concentration are shown in Supplementary Figure 1. After Bonferroni's correction, 25 and seven SNPs were significantly [*P* < 1.21 × 10^−6^ (0.05/41,352)] associated with eGFR (Table 1) or the serum concentration of creatinine (Table 2), respectively. None of these SNPs was associated with both eGFR and serum creatinine concentration.

**Table 1:** The 25 SNPs significantly (*P* < 1.21 × 10^−^^6^) associated with eGFR in the EWAS

Gene	dbSNP	Nucleotide (amino acid) substitution^a^	Chromosome: position	MAF (%)	*P* (genotype)
*GGCT*	rs115910467	C/T (R108H)	7: 30497200	8.2	8.10 × 10^−17^
*COL6A5*	rs200982668	G/A (E2501K)	3: 130470894	1.3	2.92 × 10^−15^
*MOB3C*	rs139537100	C/T (R24Q)	1: 46615006	1.2	1.19 × 10^−14^
*CXCL8*	rs188378669	G/T (E31*)	4: 73741568	1.2	1.44 × 10^−14^
*PLCB2*	rs200787930	C/T (E1095K)	15: 40289298	1.2	1.80 × 10^−14^
*MARCH1*	rs61734696	G/T (Q137K)	4: 164197303	1.2	2.05 × 10^−14^
*VPS33B*	rs199921354	C/T (R80Q)	15: 91013841	1.2	2.86 × 10^−14^
*TMOD4*	rs115287176	G/A (R277W)	1: 151170961	1.2	3.06 × 10^−14^
*TNC*	rs138406927	C/T (A1096T)	9: 115064848	2.1	3.05 × 10^−13^
*ZNF77*	rs146879198	G/A (R340*)	19: 2934109	1.2	4.70 × 10^−13^
*COL6A3*	rs146092501	C/T (E1386K)	2: 237371861	1.2	5.91 × 10^−13^
*ADGRL3*	rs192210727	G/T (R580I)	4: 61909615	1.3	6.67 × 10^−12^
*C21orf59*	rs76974938	C/T (D67N)	21: 32609946	2.4	2.44 × 10^−11^
*KRR1*	rs17115182	G/A (P43S)	12: 75508405	7.0	5.31 × 10^−11^
*PTCH2*	rs147284320	C/T (V503I)	1: 44828589	2.0	2.28 × 10^−9^
*MUC17*	rs78010183	A/T (T1305S)	7: 101035329	1.8	3.82 × 10^−9^
*SCN10A*	rs77804526	C/T (V1697I)	3: 38698131	0.1	6.83 × 10^−8^
*RFTN1*	rs180950245	C/G (N439K)	3: 16323391	0.1	1.63 × 10^−7^
*IGSF9B*	rs201459911	G/A (A1115V)	11: 133920381	0.7	2.14 × 10^−7^
*IQSEC3*	rs12822449	T/C (S283P)	12: 125856	0.2	3.51 × 10^−7^
*CCDC186*	rs79637542	C/T (A771T)	10: 114127543	0.2	5.10 × 10^−7^
*PRAMEF12*	rs199576535	G/A (V341I)	1: 12777168	1.0	5.91 × 10^−7^
	rs1873059	G/A	11: 45519053	47.4	6.95 × 10^−7^
*PTCHD3*	rs77473776	T/G (K186Q)	10: 27413695	30.6	9.41 × 10^−7^
*L1TD1*	rs2886644	C/T (T613I)	1: 62210612	11.0	1.04 × 10^−6^

The relation of genotypes of SNPs to eGFR was evaluated by linear regression analysis. ^a^Major allele/minor allele.

**Table 2:** The seven SNPs significantly (*P* < 1.21 × 10^−^^6^) associated with the serum concentration of creatinine in the EWAS

Gene	dbSNP	Nucleotide (amino acid) substitution^a^	Chromosome: position	MAF (%)	*P* (genotype)
*CAT*	rs139421991	G/A (R320Q)	11: 34456720	0.3	6.34 × 10^−11^
*EIF2AK4*	rs35602605	G/T (G1306C)	15: 40016658	0.1	1.60 × 10^−10^
*SP7*	rs188929035	G/A (A5V)	12: 53329374	0.4	7.10 × 10^−9^
*CSMD2*	rs148658404	G/A (S3311F)	1: 33533855	0.7	3.89 × 10^−7^
*SASH1*	rs199980930	G/A	6: 148546146	0.1	4.57 × 10^−7^
*RNF123*	rs35620248	G/A (R387Q)	3: 49700521	0.3	1.20 × 10^−6^
*ALG12*	rs3922872	T/C (I393V)	22: 49904240	5.5	1.20 × 10^−6^

The relation of genotypes of SNPs to the serum concentration of creatinine was evaluated by linear regression analysis. ^a^Major allele/minor allele.

We next examined the relation of genotypes for 41,372 SNPs that passed quality control to the serum concentration of uric acid in 9934 subjects not taking uric acid-lowering medications by linear regression analysis. A Manhattan plot for this EWAS is also shown in Supplementary Figure 1. After Bonferroni's correction, six SNPs were significantly [*P* < 1.21 × 10^−6^ (0.05/41,372)] associated with the serum concentration of uric acid (Table 3).

**Table 3:** The six SNPs significantly (*P* < 1.21 × 10^−^^6^) associated with the serum concentration of uric acid in the EWAS

Gene	dbSNP	Nucleotide (amino acid) substitution^a^	Chromosome: position	MAF (%)	*P* (genotype)
*SLC22A12*	rs121907892	G/A (W258*)	11: 64593747	2.4	1.23 × 10^−130^
*ATG2A*	rs188780113	G/A (R478C)	11: 64911072	3.3	1.00 × 10^−39^
*SLC22A12*	rs505802	G/A	11: 64589600	17.5	1.27 × 10^−24^
*CDC42BPG*	rs55975541	G/A (R1237W)	11: 64829729	16.5	3.90 × 10^−13^
*SLC2A9*	rs3775948	G/C	4: 9993558	42.4	1.40 × 10^−12^
*SLC2A9*	rs3733591	T/C (H265R)	4: 9920506	28.7	1.01 × 10^−6^

The relation of genotypes of SNPs to the serum concentration of uric acid was evaluated by linear regression analysis ^a^Major allele/minor allele.

### EWASs for CKD and hyperuricemia

We performed an EWAS for CKD with 5161 subjects [3270 individuals with CKD (eGFR of <60 mL min^−1^ 1.73 m^−2^), 1891 controls (eGFR of ≥90 mL min^−1^ 1.73 m^−2^)] (Table 4). Age, the frequency of men, body mass index, and the prevalence of hypertension, diabetes mellitus, dyslipidemia, and hyperuricemia as well as systolic blood pressure, fasting plasma glucose level, blood glycosylated hemoglobin (hemoglobin A_1c_) content, and serum concentrations of triglycerides and uric acid were greater, whereas the serum concentration of high density lipoprotein (HDL)–cholesterol and hemoglobin concentration were lower, in subjects with CKD than in controls.

**Table 4:** Characteristics of the 5161 study subjects in the EWAS for CKD

Characteristic	CKD	Controls	*P*
No. of subjects	3270	1891	
Age (years)	69.9 ± 10.9	53.2 ± 13.4	<0.0001
Sex (male/female, %)	61.4/38.6	52.6/47.4	<0.0001
Body mass index (kg/m^2^)	23.5 ± 3.5	23.0 ± 3.7	<0.0001
Hypertension (%)	75.3	43.2	<0.0001
Systolic blood pressure (mmHg)	140 ± 26	128 ± 24	<0.0001
Diastolic blood pressure (mmHg)	77 ± 15	76 ± 14	0.0111
Diabetes mellitus (%)	38.4	25.4	<0.0001
Fasting plasma glucose (mmol/L)	6.76 ± 2.93	6.39 ± 2.81	<0.0001
Blood hemoglobin A_1c_ (%)	6.09 ± 1.31	6.06 ± 1.47	<0.0001
Dyslipidemia (%)	69.0	56.4	<0.0001
Serum triglycerides (mmol/L)	1.58 ± 0.99	1.34 ± 1.15	<0.0001
Serum HDL-cholesterol (mmol/L)	1.37 ± 0.45	1.54 ± 0.47	<0.0001
Serum LDL-cholesterol (mmol/L)	3.06 ± 0.90	3.06 ± 0.85	0.7907
Hyperuricemia (%)	37.0	7.9	<0.0001
Serum uric acid (μmol/L)	374 ± 105	292 ± 84	<0.0001
Hemoglobin (g/dL)	13.3 ± 1.6	13.6 ± 1.5	<0.0001
Blood urea nitrogen (mmol/L)	7.54 ± 4.41	4.67 ± 1.39	<0.0001
Serum creatinine (μmol/L)	130 ± 161	51 ± 9	<0.0001
eGFR (mL min^−1^ 1.73 m^−2^)	47.4 ± 13.2	102.9 ± 18.9	<0.0001

Quantitative data are means ± SD and were compared between subjects with CKD and controls with the Mann-whitney *u* test. Categorical data were compared between the two groups with the pearson's chi-square test. Based on Bonferroni's correction, a *P* value of <0.0028 (0.05/18) was considered statistically significant. HDL, high density lipoprotein; LDL, low density lipoprotein.

We examined the relation of allele frequencies of 41,352 SNPs to CKD with Fisher's exact test. A Manhattan plot for the EWAS of CKD is shown in Supplementary Figure 2. After Bonferroni's correction, 49 SNPs were significantly [*P* < 1.21 × 10^−6^ (0.05/41,352)] associated with CKD (Supplementary Table 1). The genotype distributions for these SNPs were in Hardy-Weinberg equilibrium (*P* > 0.001) among both subjects with CKD and controls (Supplementary Table 2).

The relation of the 49 SNPs identified by the EWAS to CKD was examined further by multivariable logistic regression analysis with adjustment for age, sex, and the prevalence of hypertension and diabetes mellitus (Supplementary Table 3). Four SNPs (rs707926 of *VARS*, rs76974938 of *C21orf59*, rs112311672 of *HDAC10*, rs41272317 of *ACAD11*) were related (*P* < 0.05 in at least one genetic model) to CKD, although there was no SNP significantly [*P* < 2.55 × 10^−4^ (0.05/196)] associated with this condition (Table 5). The minor alleles of these SNPs were all risk factors for CKD. The rs76974938 [C/T (D67N)] SNP of *C21orf59* was associated with both eGFR and CKD.

**Table 5:** Relation of SNPs to CKD as determined by multivariable logistic regression analysis

SNP		Dominant		Recessive		Additive 1		Additive 2	
		*P*	OR (95% CI)	*P*	OR (95% CI)	*P*	OR (95% CI)	*P*	OR (95% CI)
rs707926	G/A	0.4239		0.0042	1.54 (1.14–2.08)	0.9753		0.0052	1.53 (1.14–2.09)
rs76974938	C/T (D67N)	0.0420	1.52 (1.00–2.42)	ND		0.0420	1.52 (1.00–2.42)	ND	
rs112311672	G/A (T398M)	0.0492	3.33 (1.00–13.35)	ND		0.0492	3.33 (1.00–13.35)	ND	
rs41272317	C/A	0.2694		0.0220	1.11 × 10^8^ (ND)	0.2094		0.0224	1.10 × 10^8^ (ND)

Multivariable logistic regression analysis was performed with adjustment for age, sex, and the prevalence of hypertension and diabetes mellitus. Based on Bonferroni's correction, a *P* value of <2.55 × 10^−4^ (0.05/196) was considered statistically significant. OR, odds ratio; CI, confidence interval; ND, not determined.

We next performed an EWAS for hyperuricemia with 11,686 subjects (2045 individuals with hyperuricemia, 9641 controls), the characteristics of whom are shown in Table 6. Age, the frequency of men, body mass index, and the prevalence of smoking, hypertension, diabetes mellitus, dyslipidemia, and CKD were greater in subjects with hyperuricemia than in controls.

**Table 6:** Characteristics of the study subjects in the EWAS for hyperuricemia

Characteristic	Hyperuricemia	Controls	*P*
No. of subjects	2045	9641	
Age (years)	59.6 ± 12.2	58.3 ± 13.2	<0.0001
Sex (male/female, %)	86.7/13.4	52.9/47.1	<0.0001
Body mass index (kg/m^2^)	24.3 ± 3.7	23.1 ± 3.4	<0.0001
Current or former smoker (%)	53.7	34.7	<0.0001
Hypertension (%)	68.9	47.2	<0.0001
Diabetes mellitus (%)	31.5	23.0	<0.0001
Dyslipidemia (%)	77.6	58.4	<0.0001
CKD (%)	44.8	17.9	<0.0001
Serum uric acid (μmol/L)	453 ± 83	296 ± 67	<0.0001

Quantitative data are means ± SD and were compared between subjects with hyperuricemia and controls with the Mann-whitney *u* test. Categorical data were compared between the two groups with the pearson's test chi-square test. Based on Bonferroni's correction, a *P* value of <0.0056 (0.05/9) was considered statistically significant.

We examined the relation of allele frequencies of 41,372 SNPs to hyperuricemia with Fisher's exact test. A Manhattan plot for the EWAS is shown in Supplementary Figure 2. After Bonferroni's correction, 35 SNPs were significantly [*P* < 1.21 × 10^−6^ (0.05/41,372)] associated with hyperuricemia (Supplementary Table 4). The genotype distributions for these SNPs were in Hardy-Weinberg equilibrium (*P* > 0.001) among both subjects with hyperuricemia and controls (Supplementary Table 5).

The relation of the 35 SNPs identified by the EWAS to hyperuricemia was examined further by multivariable logistic regression analysis with adjustment for age and sex (Supplementary Table 6). Three SNPs (rs115445569 of *ACOT11*, rs116911833 of *TRIM7*, rs60854092 of *NOTCH2*) were related (*P* < 0.05 in at least one genetic model) to hyperuricemia, although there was no SNP significantly [*P* < 3.57 × 10^−4^ (0.05/140)] associated with this condition (Table 7). The minor *T* and *A* alleles of rs115445569 and rs116911833, respectively, were risk factors for hyperuricemia, whereas the minor *A* allele of rs60854092 was protective against hyperuricemia.

**Table 7:** Relation of SNPs to hyperuricemia as determined by multivariable logistic regression analysis

SNP		Dominant		Recessive		Additive 1		Additive 2	
		*P*	OR (95% CI)	*P*	OR (95% CI)	*P*	OR (95% CI)	*P*	OR (95% CI)
rs115445569	C/T (R64Q)	0.0228	1.43 (1.05–1.93)	0.5153		0.0266	1.42 (1.04–1.92)	0.5114	
rs116911833	G/A (T80M)	0.2547		0.0331	7.21 (1.19–43.73)	0.1557		0.0337	7.15 (1.18–43.43)
rs60854092	T/A (F1689I)	0.0377	0.83 (0.69–0.99)	0.4797		0.0455	0.84 (0.70–1.00)	0.4628	

Multivariable logistic regression analysis was performed with adjustment for age and sex. Based on Bonferroni's correction, a *P* value of <3.57 × 10^−4^ (0.05/140) was considered statistically significant.

### Relation of identified SNPs to eGFR or serum concentrations of creatinine or uric acid

We examined the relations of genotypes for SNPs identified in the various EWASs to eGFR or serum concentrations of creatinine or uric acid by one-way analysis of variance (ANOVA). The 25 SNPs identified in the EWAS for eGFR were all significantly [*P* < 0.0018 (0.05/28)] associated with eGFR (Table 8). Among the four SNPs identified in the analysis of CKD, only rs76974938 was significantly related to eGFR. The seven SNPs identified in the EWAS for serum creatinine concentration were all significantly [*P* < 0.0045 (0.05/11)] related to the serum concentration of creatinine (Table 9). Among the four SNPs identified in the analysis of CKD, only rs76974938 was significantly related to the serum concentration of creatinine. The six SNPs identified in the EWAS for serum uric acid concentration were all significantly [*P* < 0.0056 (0.05/9)] related to the serum concentration of uric acid, but the three SNPs identified in the analysis of hyperuricemia were not (Table 10).

**Table 8:** Relation of SNPs identified in the present study to eGFR

SNP		eGFR (mL min^−1^ 1.73 m^−2^)			*P*
Associated with eGFR and CKD					
rs76974938	C/T (D67N)	*CC*	*CT*		
		73.2 ± 21.3	67.2 ± 13.1		**2.44 × 10^−11^**
Associated with eGFR					
rs115910467	C/T (R108H)	*CC*	*CT*		
		73.2 ± 21.2	66.3 ± 13.9		**8.10 × 10^−17^**
rs200982668	G/A (E2501K)	*GG*	*GA*		
		72.0 ± 20.6	80.8 ± 15.5		**2.92 × 10^−15^**
rs139537100	C/T (R24Q)	*CC*	*CT*		
		72.0 ± 20.6	80.8 ± 15.5		**1.19 × 10^−14^**
rs188378669	G/T (E31*)	*GG*	*GT*		
		72.0 ± 20.6	80.9 ± 15.5		**1.44 × 10^−14^**
rs200787930	C/T (E1095K)	*CC*	*CT*		
		72.0 ± 20.6	80.8 ± 15.5		**1.80 × 10^−14^**
rs61734696	G/T (Q137K)	*GG*	*GT*		
		72.0 ± 20.6	80.8 ± 15.4		**2.05 × 10^−14^**
rs199921354	C/T (R80Q)	*CC*	*CT*		
		72.0 ± 20.6	80.8 ± 15.5		**2.86 × 10^−14^**
rs115287176	G/A (R277W)	*GG*	*GA*		
		72.0 ± 20.6	80.8 ± 15.4		**3.06 × 10^−14^**
rs138406927	C/T (A1096T)	*CC*	*CT*		
		73.1 ± 21.3	66.2 ± 14.5		**3.05 × 10^−13^**
rs146879198	G/A (R340*)	*GG*	*GA*		
		72.0 ± 20.6	80.5 ± 15.4		**4.70 × 10^−13^**
rs146092501	C/T (E1386K)	*CC*	*CT*		
		72.0 ± 20.6	80.4 ± 15.5		**5.91 × 10^−13^**
rs192210727	G/T (R580I)	*GG*	*GT*	*TT*	
		72.0 ± 20.6	79.6 ± 16.0	84.6 ± 13.6	**5.64 × 10^−11^**
rs17115182	G/A (P43S)	*GG*	*GA*		
		73.2 ± 21.3	66.7 ± 13.3		**5.31 × 10^−11^**
rs147284320	C/T (V503I)	*CC*	*CT*		
		73.4 ± 17.5	78.6 ± 15.9		**2.28 × 10^−9^**
rs78010183	A/T (T1305S)	*AA*	*AT*		
		72.1 ± 20.7	77.4 ± 15.3		**3.82 × 10^−9^**
rs77804526	C/T (V1697I)	*CC*	*CT*	*TT*	
		72.2 ± 20.0	100.0 ± 116.9	65.1	**9.50 × 10^−9^**
rs180950245	C/G (N439K)	*CC*	*CG*		
		72.2 ± 20.0	92.5 ± 99.1		**1.23 × 10^−7^**
rs201459911	G/A (A1115V)	*GG*	*GA*		
		72.4 ± 20.5	65.1 ± 22.0		**2.14 × 10^−7^**
rs12822449	T/C (S283P)	*TT*	*TC*		
		70.5 ± 21.7	50.6 ± 17.8		**3.51 × 10^−7^**
rs79637542	C/T (A771T)	*CC*	*CT*		
		72.2 ± 20.0	86.2 ± 71.1		**5.10 × 10^−7^**
rs199576535	G/A (V341I)	*GG*	*GA*		
		72.1 ± 20.6	78.2 ± 14.1		**5.91 × 10^−7^**
rs1873059	G/A	*GG*	*GA*	*AA*	
		71.2 ± 19.4	72.2 ± 21.2	73.8 ± 20.1	**3.45 × 10^−6^**
rs77473776	T/G (K186Q)	*TT*	*TG*	*GG*	
		72.9 ± 21.7	72.1 ± 19.5	69.0 ± 15.0	**2.89 × 10^−7^**
rs2886644	C/T (T613I)	*CC*	*CT*	*TT*	
		73.3 ± 21.4	70.9 ± 18.6	68.3 ± 18.1	**6.53 × 10^−6^**
Associated with CKD					
rs707926	G/A	*GG*	*GA*	*AA*	
		72.3 ± 20.4	72.6 ± 20.9	70.5 ± 18.3	0.0255
rs112311672	G/A (T398M)	*GG*	*GA*		
		72.3 ± 20.5	72.4 ± 15.1		0.9683
rs41272317	C/A	*CC*	*CA*	*AA*	
		72.3 ± 20.5	72.6 ± 20.2	73.5 ± 5.0	0.8680

Data were compared among genotypes by one-way ANOVA. Based on Bonferroni's correction, *P* values of <0.0018 (0.05/28) were considered statistically significant and are shown in bold.

**Table 9:** Relation of SNPs identified in the present study to serum creatinine concentration

SNP		Serum creatinine (μmol/L)			*P*
Associated with serum creatinine					
rs139421991	G/A (R320Q)	*GG*	*GA*		
		76.7 ± 76.7	129.9 ± 222.4		**6.34 × 10^−11^**
rs35602605	G/T (G1306C)	*GG*	*GT*	*TT*	
		78.1 ± 81.7	200.2 ± 354.0	88.4	**4.50 × 10^−11^**
rs188929035	G/A (A5V)	*GG*	*GA*	*AA*	
		78.1 ± 81.1	98.7 ± 161.1	1502.8	**<1.0 × 10^−23^**
rs148658404	G/A (S3311F)	*GG*	*GA*		
		77.9 ± 78.7	110.0 ± 216.2		**3.89 × 10^−7^**
rs199980930	G/A	*GG*	*GA*		
		78.1 ± 82.1	151.1 ± 238.2		**4.57 × 10^−7^**
rs35620248	G/A (R387Q)	*GG*	*GA*		
		78.1 ± 81.2	126.0 ± 242.6		**1.23 × 10^−6^**
rs3922872	T/C (I393V)	*TT*	*TC*	*CC*	
		77.2 ± 76.9	87.7 ± 118.5	106.3 ± 204.8	**6.68 × 10^−6^**
Associated with CKD					
rs707926	G/A	*GG*	*GA*	*AA*	
		77.9 ± 81.9	79.2 ± 86.0	77.2 ± 74.4	0.6246
rs112311672	G/A (T398M)	*GG*	*GA*		
		78.4 ± 83.3	68.4 ± 14.4		0.3146
rs41272317	C/A	*CC*	*CA*	*AA*	
		78.3 ± 83.1	78.2 ± 83.3	69.7 ± 21.7	0.9127
rs76974938	C/T (D67N)	*CC*	*CT*		
		80.1 ± 89.3	66.8 ± 16.3		**0.0003**

Data were compared among genotypes by one-way ANOVA. Based on Bonferroni's correction, *P* values of <0.0045 (0.05/11) were considered statistically significant and are shown in bold.

**Table 10:** Relation of SNPs identified in the present study to the serum concentration of uric acid

SNP		Serum uric acid (μmol/L)			*P*
Associated with serum uric acid					
rs121907892	G/A (W258*)	*GG*	*GA*	*AA*	
		333 ± 93	230 ± 79	50 ± 18	**<1.0 × 10^−23^**
rs188780113	G/A (R478C)	*GG*	*GA*	*AA*	
		331 ± 93	283 ± 103	197 ± 105	**<1.0 × 10^−23^**
rs505802	G/A	*GG*	*GA*	*AA*	
		334 ± 92	318 ± 98	291 ± 97	**<1.0 × 10^−23^**
rs55975541	G/A (R1237W)	*GG*	*GA*	*AA*	
		332 ± 94	319 ± 95	306 ± 111	**3.62 × 10^−12^**
rs3775948	G/C	*GG*	*GC*	*CC*	
		335 ± 93	329 ± 94	314 ± 97	**1.37 × 10^−12^**
rs3733591	T/C (H265R)	*TT*	*TC*	*CC*	
		324 ± 94	331 ± 96	339 ± 93	**6.36 × 10^−6^**
Associated with hyperuricemia					
rs115445569	C/T (R64Q)	*CC*	*CT*	*TT*	
		328 ± 95	341 ± 101	345 ± 170	0.1012
rs116911833	G/A (T80M)	*GG*	*GA*	*AA*	
		328 ± 95	324 ± 89	352 ± 109	0.5779
rs60854092	T/A (F1689I)	*TT*	*TA*	*AA*	
		328 ± 94	325 ± 98	322 ± 121	0.5501

Data were compared among genotypes by one-way ANOVA. Based on Bonferroni's correction, *P* values of <0.0056 (0.05/9) were considered statistically significant and are shown in bold.

### Relation of SNPs identified in the present study to phenotypes previously examined in GWASs

We examined the relation of genes, chromosomal loci, and SNPs identified in the present study to phenotypes previously probed in GWASs with data available in public databases [GWAS Catalog (http://www.ebi.ac.uk/gwas) and GWAS Central (http://www.gwascentral.org/browser)]. Chromosomal region 11p11.2 and *SASHI* were previously shown to be susceptibility loci for CKD [15] or diabetic nephropathy [34], respectively. In addition, *MARCH1* and *RFTN1* were previously shown to be related to urinary uromodulin [35] or serum uric acid [36] levels, respectively. The remaining 31 SNPs identified in the present study as being related to eGFR, the serum creatinine concentration, or CKD were not previously identified as genetic determinants of renal function-related traits or CKD (Supplementary Table 7). *SLC22A12* [27, 37], *CDC42BPG* [29], and *SLC2A9* [32, 37–39] were previously implicated as determinants of the serum concentration of uric acid or gout, whereas the remaining four SNPs identified in the present study as being related to the serum concentration of uric acid or hyperuricemia were not previously found to influence serum uric acid levels, hyperuricemia, or gout (Supplementary Table 8).

## DISCUSSION

We have now shown that rs76974938 [C/T (D67N)] of *C21orf59* was associated with eGFR and CKD. Twenty-four additional SNPs and seven SNPs were significantly related to eGFR or the serum concentration of creatinine, respectively. Among these genes and loci, 11p11.2 and *SASH1* were previously identified as susceptibility loci for CKD [15] or diabetic nephropathy [34], respectively. *MARCH1* and *RFTN1* were also previously shown to be related to urinary uromodulin [35] or serum uric acid [36] levels, respectively. The remaining 21 and six SNPs identified in our study are thus potential novel loci related to eGFR or to the serum concentration of creatinine, respectively, in Japanese. We also identified three additional SNPs related to CKD, all of which are candidates for novel susceptibility loci for this condition.

We also found that rs121907892 [G/A (W258*)] and rs505802 (G/A) of *SLC22A12*, rs188780113 [G/A (R478C)] of *ATG2A*, rs55975541 [G/A (R1237W)] of *CDC42BPG*, and rs3775948 (G/C) and rs3733591 [T/C (H265R)] of *SLC2A9* were significantly associated with the serum concentration of uric acid. In addition, rs115445569 [C/T (R64Q)] of *ACOT11*, rs116911833 [G/A (T80M)] of *TRIM7*, and rs60854092 [T/A (F1689I) of *NOTCH2* were related to hyperuricemia, although the genotypes of these SNPs were not related to the serum concentration of uric acid. Among these genes, *SLC22A12* [27, 37], *CDC42BPG* [29], and *SLC2A9* [32, 37–39] were previously found to be related to serum uric acid levels or gout. The remaining four genes (*ATG2A*, *ACOT11*, *TRIM7*, and *NOTCH2*) may be novel loci that influence the serum concentration of uric acid or confer susceptibility to hyperuricemia.

### SNPs associated with renal function

The chromosome 21 open reading frame 59 gene (*C21orf59*) is located at chromosomal region 21q22.11 (NCBI Gene, https://www.ncbi.nlm.nih.gov/gene) and is widely expressed including in the kidney (The Human Protein Atlas, http://www.proteinatlas.org). Studies in zebrafish and *Xenopus* have revealed that the protein encoded by *C21orf59* activates the motility and polarization of cilia and thereby contributes to cilia-mediated processes such as the generation of fluid flow [40]. The primary cilium is a microtubule-based organelle. Impairment of ciliary function can result in polycystic kidney disease [41]. C21orf59 contributes to the assembly of dynein arms in motile cilia, with mutations in *C21orf59* having been found to cause ciliary dyskinesia [42]. We have now shown that rs76974938 [C/T (D67N)] of *C21orf59* was significantly associated with eGFR and CKD, with the minor *T* allele representing a risk factor for CKD. Given that C21orf59 is implicated in cilium function and that cilia serve as mechanosensors to detect fluid flow in the lumen of renal tubules, the association of *C21orf59* with eGFR and CKD might reflect an effect of this gene on renal tubular function.

In a previous GWAS for individuals of European ancestry [9], the MAFs of CKD-associated SNPs were 13% to 27%, their effect sizes (percentage change in serum creatinine levels) were −1.0% to 1.1%, and the allele odds ratios (ORs) for CKD were 0.84 to 1.07. Another GWAS performed with subjects of European ancestry [10] identified eGFR-associated SNPs whose MAFs were 5% to 50% and ORs for CKD were 0.80 to 1.19. In a meta-analysis of GWASs with a total of 137,629 individuals of European ancestry, the MAFs of eGFR-associated SNPs were 10% to 47% [15], and in a similar analysis with a total of 71,149 East Asian individuals the MAFs of kidney function-associated SNPs were 11% to 43% [14].

In the present study, rs76974938 of *C21orf59* had a MAF of 2.4% and an allele OR of 4.52 for CKD, and it showed a difference in eGFR of 6.0 mL min^−1^ 1.73 m^−2^ or in the serum concentration of creatinine of 13.3 μmol/L among genotypes. This SNP was thus a low-frequency variant with a moderate effect size. For the remaining 24 SNPs [MAF, difference in eGFR (mL min^−1^ 1.73 m^−2^) among genotypes] identified in the EWAS of eGFR, rs77804526 (0.1%, 27.8), rs180950245 (0.1%, 20.3), rs12822449 (0.2%, 19.9), and rs79637542 (0.2%, 14.0) were rare variants with a large effect size; rs200982668 (1.3%, 8.8), rs139537100 (1.2%, 8.8), rs188378669 (1.2%, 8.9), rs200787930 (1.2%, 8.8), rs61734696 (1.2%, 8.8), rs199921354 (1.2%, 8.8), rs115287176 (1.2%, 8.8), rs138406927 (2.1%, 6.9), rs146879198 (1.2%, 8.5), rs146092501 (1.2%, 8.4), rs192210727 (1.3%, 7.6), rs147284320 (2.0%, 5.2), rs78010183 (1.8%, 5.3), rs201459911 (0.7%, 7.3), and rs199576535 (1.0%, 6.1) were low-frequency variants with a moderate effect size; and rs115910467 (8.2%, 6.9), rs17115182 (7.0%, 6.5), rs1873059 (47.4%, 2.6), rs77473776 (30.6%, 3.9), and rs2886644 (11.0%, 5.0) were common variants with a low to moderate effect size (Supplementary Figure 3A). In the case of the seven SNPs [MAF, difference in serum creatinine concentration (μmol/L) among genotypes] identified in the EWAS for serum creatinine concentration, rs139421991 (0.3%, 53.2), rs35602605 (0.1%, 122.1), rs188929035 (0.4%, 20.6), rs199980930 (0.1%, 73.0), and rs35620248 (0.3%, 47.9) were rare variants with a moderate to large effect size; rs148658404 (0.7%, 32.1) was a low-frequency variant with a moderate effect size; and rs3922872 (5.5%, 10.5) was a common variant with a small effect size (Supplementary Figure 3B).

### SNPs associated with the serum concentration of uric acid

The autophagy related 2A gene (*ATG2A*) is located at chromosomal region 11q13.1 (NCBI Gene) and is expressed in various organs including the kidney and gastrointestinal tract (The Human Protein Atlas). Autophagy is an intracellular process in which cytoplasmic material is enveloped by authophagosomes and delivered to lysosomes for degradation [43]. Mammalian ATG2A is localized to the autophagosome membrane as well as to lipid droplets. Knockdown of both ATG2A and ATG2B in adipocytes results in an increase in the size and number of lipid droplets and in their clustering. The mammalian proteins thus promote autophagosome formation [44]. Activation of neutrophils in gout promotes the formation of proinflammatory neutrophil extracellular traps, a process linked to autophagy-related signaling and interleukin-1β activity [45]. We have now shown that rs188780113 [G/A (R478C)] of *ATG2A* was significantly associated with the serum concentration of uric acid, with the minor *A* allele being related to decreased uric acid levels. Although the molecular mechanism underlying this association remains unclear, it might be attributable to the role of ATG2A in autophagy-related metabolic processes.

The acyl-CoA thioesterase 11 gene (*ACOT11*) is located at chromosomal region 1p32.3 (NCBI Gene) and is expressed in various organs including the kidney and gastrointestinal tract (The Human Protein Atlas). Mammalian acyl-CoA thioesterases catalyze the conversion of activated fatty acids to the corresponding nonesterified fatty acid and coenzyme A [46]. *ACOT11* mRNA levels were increased in mouse brown adipose tissue by cold exposure and decreased by warm temperatures as well as were higher in this tissue of obesity-resistant mice than in obesity-prone mice [47]. ACOT11 functions to reduce energy consumption and conserve calories, also suggestive of a role in obesity [48]. In nutritional excess, the overproduction of free fatty acids by ACOT11 induces insulin resistance, leading to increases in inflammation and endoplasmic reticulum stress [48]. We have now shown that rs115445569 [C/T (R64Q)] of *ACOT11* was related to hyperuricemia, with the minor *T* allele representing a risk factor this condition. The association of *ACOT11* with hyperuricemia might reflect the fact that ACOT11 is implicated in adipose tissue metabolism and that obesity is a risk factor for hyperuricemia.

The tripartite motif containing 7 gene (*TRIM7*) is located at chromosomal region 5q35.3 (NCBI Gene) and is widely expressed including in the kidney and gastrointestinal tract (The Human Protein Atlas). A family of tripartite motif (TRIM) proteins comprises >70 members and contributes to various biological processes [49–54]. TRIM7 is a glycogenin-interacting protein that contributes to cellular glucose metabolism [55]. We have now shown that rs116911833 [G/A (T80M)] of *TRIM7* was significantly associated with hyperuricemia, with the minor *A* allele representing a risk factor for this condition. Given the potential role of TRIM7 in diverse cellular processes, the molecular mechanism underlying this association remains unclear.

The *NOTCH2* gene is located at chromosomal region 1p12 (NCBI Gene) and is ubiquitously expressed including in the kidney and gastrointestinal tract (The Human Protein Atlas). Notch2 functions as a receptor for membrane-bound ligands and is implicated in the development of vessels, kidney, and liver [56]. It also contributes to skeletal homeostasis, osteoblastogenesis, and osteoclastogenesis [57]. Mutations in *NOTCH2* that result in the production of a stable truncated protein are responsible for Hajdu-Cheney syndrome, which is characterized by defects in craniofacial development, osteoporosis with fractures, acro-osteolysis, neurological complications, cardiovascular defects, and polycystic kidneys [58]. Mutations of *NOTCH2* also cause Alagille syndrome [59]. *NOTCH2* was shown to be a susceptibility locus for type 2 diabetes mellitus [60]. We have now shown that rs60854092 [T/A (F1689I)] of *NOTCH2* was significantly associated with hyperuricemia, with the minor *A* allele being protective against this condition. Given that insulin resistance may be a common underlying mechanism contributing to type 2 diabetes mellitus and hyperuricemia [61], the association of *NOTCH2* with hyperuricemia might reflect the effect of this gene on type 2 diabetes.

In a previous large-scale GWAS for serum uric acid concentration [30], the MAFs of related SNPs were 11% to 49% and their effect sizes ranged from −10.9 to 22.2 μmol/L (−0.184 to 0.373 mg/dL). In our study, among the six SNPs significantly related to the serum concentration of uric acid, the MAF (difference in serum uric acid level among genotypes) of rs121907892 or rs188780113 was 2.4% (283 μmol/L) and 3.3% (134 μmol/L), respectively. These SNPs were thus low-frequency variants with a large effect size. The MAF (difference in serum uric acid level among genotypes) of the remaining four SNPS (rs505802, rs55975541, rs3775948, and rs3733591) was 17.5% (43 μmol/L), 16.5% (26 μmol/L), 42.4% (21 μmol/L), and 28.7% (15 μmol/L), respectively. These SNPs were thus common variants with a small to moderate effect size (Supplementary Figure 4). The MAFs (allele ORs) of the three SNPs related to hyperuricemia (rs115445569, rs116911833, and rs60854092) were 1.1% (1.51), 2.0% (1.21), and 4.6% (0.85), respectively. These SNPs were thus low-frequency variants with a small to moderate effect size.

### Study limitations

There are several limitations in the present study. (i) Although an eGFR is affected by body weight, especially skeletal muscle mass, the formula for eGFR used in the present study did not include body weight. (ii) We did not obtain information on microhematuria or microalbuminuria in controls. Information by detailed clinical examination including renal biopsy was not obtained in most subjects, given that such diagnostic procedures were not considered feasible for an epidemiological study whose subjects were recruited from the general population. (iii) Some patients were diagnosed for CKD with an eGFR <60 mL min^−1^ 1.73 m^−2^ at one point of examination. Dehydration status in such subjects was not examined. Data for the rate of GFR fall in a long-term follow up were not available for the present study subjects. (iv) Given that our results were not replicated, they will require validation in other subject panels or ethnic groups. (v) It is possible that SNPs identified in the present study are in linkage disequilibrium with other polymorphisms in nearby genes that are actually responsible for the observed associations. (vi) Three SNPs associated with CKD were not related to eGFR or to the serum concentration of creatinine. In addition, the three SNPs associated with hyperuricemia were not related to the serum concentration of uric acid. It is possible that these discrepancies are attributable to the effects of medical treatment. (vii) The molecular mechanisms of the observed associations remain to be determined.

## CONCLUSIONS

In conclusion, we have identified rs76974938 [C/T (D67N)] of *C21orf59* as a novel genetic determinant of renal function and CKD. We identified an additional 21 and six SNPs as novel determinants of eGFR or the serum concentration of creatinine, respectively, as well as an additional three SNPs as novel candidate susceptibility loci for CKD. We also identified rs188780113 [G/A (R478C)] of *ATG2A* as a novel determinant of the serum concentration of uric acid as well as rs115445569 of *ACOT11*, rs116911833 of *TRIM7*, and rs60854092 of *NOTCH2* as potential novel susceptibility loci for hyperuricemia. Analysis of these SNPs may be informative for assessment of the genetic risk for CKD or hyperuricemia in Japanese.

## MATERIALS AND METHODS

### Study subjects

For the EWASs of eGFR or the serum concentration of creatinine, a total of 12,565 Japanese was examined. A total of 9934 subjects who did not take uric acid-lowering medications was examined in the EWAS for the serum concentration of uric acid. In the EWASs for CKD or hyperuricemia, 5161 individuals (3270 subjects with CKD, 1891 controls) or 11,686 individuals (2045 subjects with hyperuricemia, 9641 controls) were examined, respectively. Study subjects were recruited as previously described [62].

Glomerular filtration rate was estimated with the use of the simplified prediction equation derived from the modified version of that described in the Modification of Diet in Renal Disease (MDRD) Study, as proposed by the Japanese Society of Nephrology [63]: eGFR (mL min^−1^ 1.73 m^−2^) = 194 × [age (years)]^−0.287^ × [serum creatinine (mg/dL)]^−1.094^ × [0.739 if female]. The National Kidney Foundation–Kidney Disease Outcomes Quality Initiative guidelines recommend a diagnosis of CKD if eGFR is <60 mL min^−1^ 1.73 m^−2^ [4]. Nonlinear relations between GFR and the risk of adverse events, such as death, cardiovascular events, and hospitalization, have been demonstrated, with an increased risk being associated with an eGFR of <60 mL min^−1^ 1.73 m^−2^ and the risk markedly rising further when values fall below 45 mL min^−1^ 1.73 m^−2^ [64]. We thus adopted the criterion of an eGFR of <60 mL min^−1^ 1.73 m^−2^ (actual range, 2.5 to 59.9 mL min^−1^ 1.73 m^−2^) for the diagnosis of CKD in the present study. The control individuals for the EWAS of CKD had an eGFR of ≥90 mL min^−1^ 1.73 m^−2^ (actual range, 90 to 584.9 mL min^−1^ 1.73 m^−2^) and did not appear to have functional or structural abnormalities of the kidneys or a history of renal disease.

Hyperuricemia was defined as a serum uric acid concentration of >416 μmol/L (actual range, 422 to 1172 μmol/L) or the taking of uric acid-lowering medication. Individuals taking drugs that may cause secondary hyperuricemia were excluded. The control individuals for the EWAS of hyperuricemia had a serum uric acid concentration of ≤416 μmol/L (actual range, 36 to 416 μmol/L) and had no history of hyperuricemia or gout or of taking uric acid-lowering medication. Autopsy cases were excluded from controls for the EWASs of both CKD and hyperuricemia.

The study protocol complied with the Declaration of Helsinki and was approved by the Committees on the Ethics of Human Research of Mie University Graduate School of Medicine, Hirosaki University Graduate School of Medicine, Tokyo Metropolitan Institute of Gerontology, and participating hospitals. Written informed consent was obtained from each participant or families of the deceased subjects.

### EWASs

Methods for collection and extraction of genomic DNA samples were described previously [62]. All EWASs (Supplementary Figure 5) were performed with the use of the HumanExome-12 v1.1 or v1.2 DNA Analysis BeadChip or Infinium Exome-24 v1.0 BeadChip (Illumina, San Diego, CA, USA). Detailed information of these exome arrays and methods of quality control were described previously [62]. Totals of 41,352 SNPs (eGFR, serum creatinine concentration, CKD) or 41,372 SNPs (serum uric acid concentration, hyperuricemia) that passed quality control were analyzed.

### Statistical analysis

The relation of genotypes of SNPs to eGFR or the serum concentrations of creatinine or uric acid in the EWAS was evaluated by linear regression analysis. In comparisons of characteristics between subjects with CKD or hyperuricemia and corresponding controls, quantitative data were compared between two groups by the Mann–Whitney U-test, given that variables showed skewed distribution (*P* <0.01 by the Kolmogorov-Smirnov and Lilliefors test). Categorical data were compared between two groups by the Pearson's chi-square test. Allele frequencies were estimated by the gene counting method, and Fisher's exact test was applied to identify departure from Hardy-Weinberg equilibrium. The relation of allele frequencies of SNPs to CKD or hyperuricemia in the EWAS was examined with Fisher's exact test. To compensate for multiple comparisons of genotypes with eGFR or the serum concentrations of creatinine or uric acid, or of allele frequencies with CKD or hyperuricemia, we applied Bonferroni's correction for statistical significance of association. Given that 41,352 or 41,372 SNPs were analyzed, the significance level was set at *P* < 1.21 × 10^−6^ (0.05/41,352 or 0.05/41,372) for the EWASs. Quantile-quantile plots either for *P* values of genotypes in the EWASs for eGFR or the serum concentrations of creatinine or uric acid or for those of allele frequencies in the EWASs for CKD or hyperuricemia are shown in Supplementary Figures 6 and 7, respectively. The inflation factor (λ) was 1.05 for eGFR, 1.06 for the serum concentration of creatinine, 1.05 for the serum concentration of uric acid, 1.17 for CKD, and 1.32 for hyperuricemia. Multivariable logistic regression analysis was performed with CKD as a dependent variable and independent variables including age, sex (0, woman; 1, man), the prevalence of hypertension and diabetes mellitus (0, no history of these conditions; 1, positive history), and genotype of each SNP. Similar analysis was performed with hyperuricemia as a dependent variable and independent variables including age, sex, and genotype of each SNP. A detailed method of analysis was described previously [62]. The relation of genotypes of isolated SNPs to eGFR or the serum concentrations of creatinine or uric acid was examined by one-way ANOVA. Bonferroni's correction was also applied to other statistical analysis as indicated. Statistical tests were performed with JMP Genomics version 6.0 software (SAS Institute, Cary, NC USA).

## SUPPLEMENTARY MATERIALS FIGURES AND TABLES














