# Supplementary material for: Identification of C21orf59 and ATG2A as novel determinants of renal function-related traits in Japanese by exome-wide association studies
Source: Oncotarget. 2017 Mar 30;8(28):45259–73. doi: 10.18632/oncotarget.16696 (PMC5542184; doi:10.18632/oncotarget.16696)
Supplement: Supplementary file 1 [file oncotarget-08-45259-s001.pdf]

Identification of *C21orf59* and *ATG2A* as novel determinants of renal function-related traits in Japanese by exome-wide association studies

SUPPLEMENTARY FIGURES AND TABLES

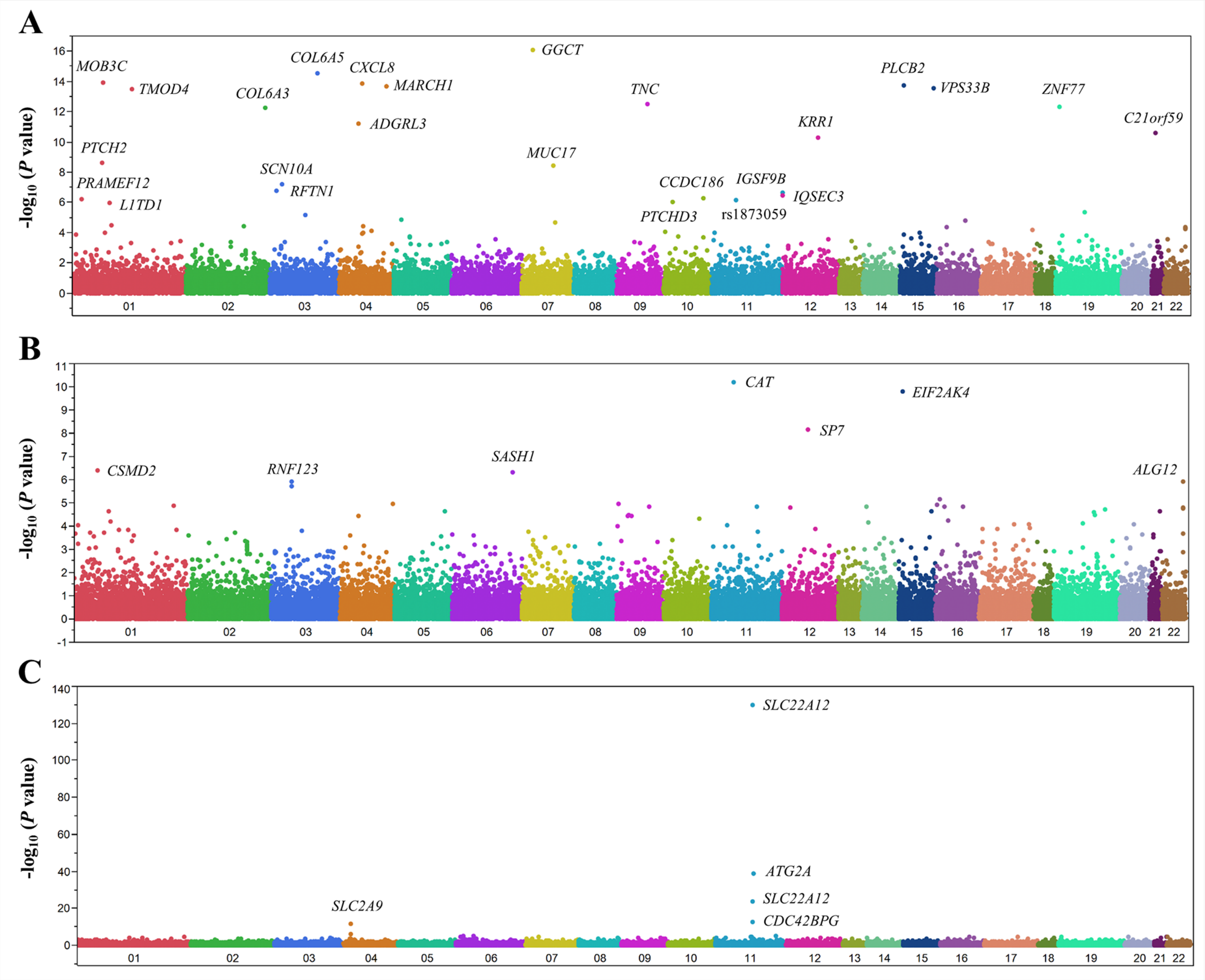

Supplementary Figure 1: Manhattan plots for *P* values of genotypes in the EWASs for eGFR **(A)** or the serum concentrations of creatinine **(B)** or uric acid **(C)**. The *P* values are shown as $-\log_{10}(P)$ on the *y*-axis with respect to the physical chromosomal position of the corresponding SNP on the *x*-axis. SNPs or the corresponding genes identified in the EWASs are indicated.

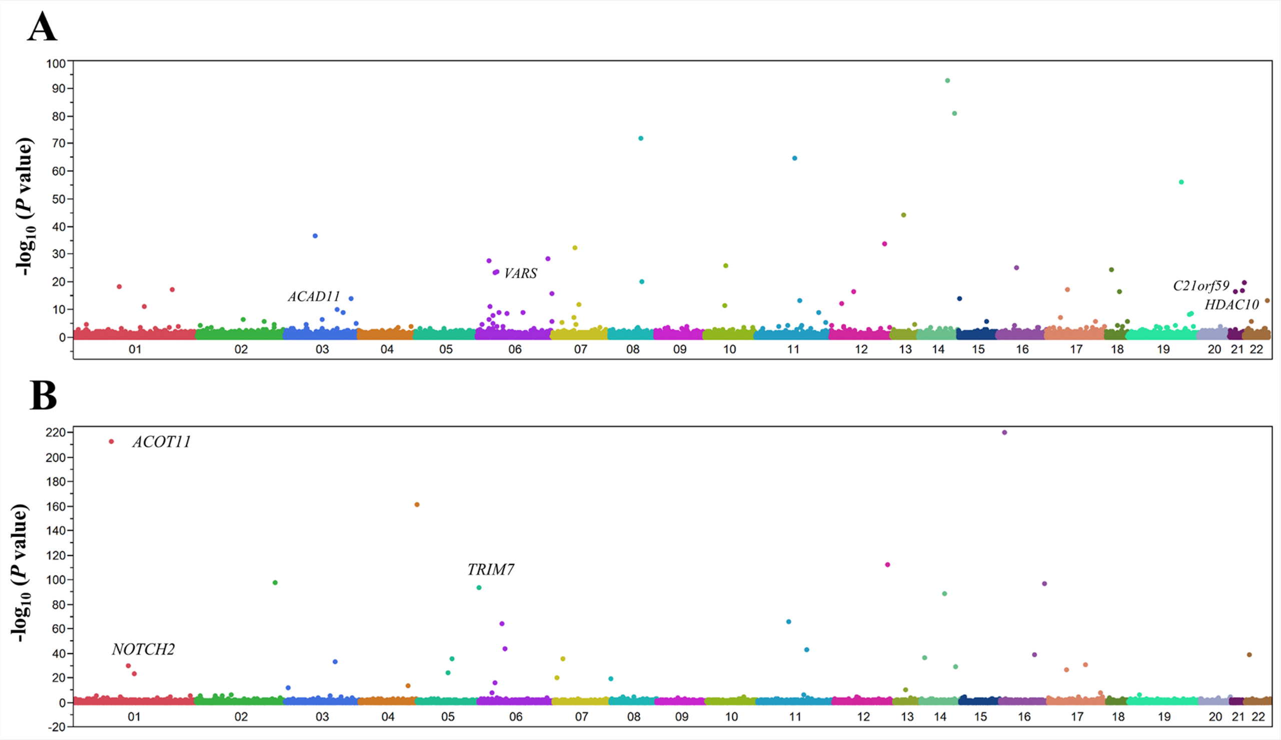

Supplementary Figure 2: Manhattan plots for *P* values of allele frequencies in the EWASs for CKD **(A)** or hyperuricemia **(B)**. The *P* values are shown as $-\log_{10}(P)$ on the *y*-axis with respect to the physical chromosomal position of the corresponding SNP on the *x*-axis. Genes identified by the EWASs and multivariable logistic regression analysis are indicated.

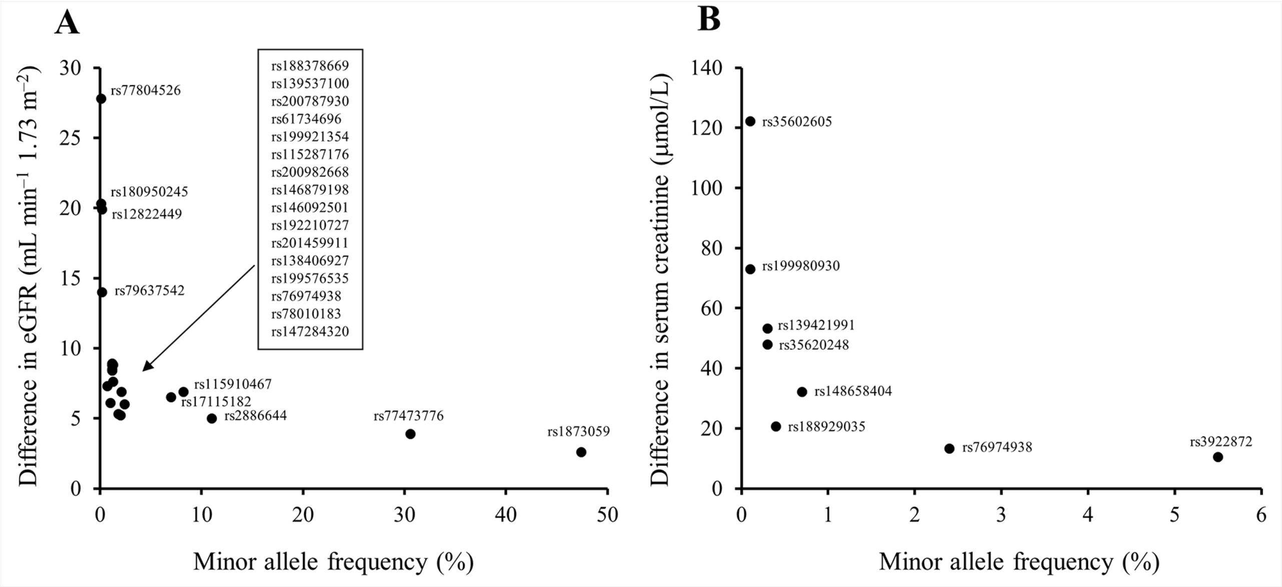

Supplementary Figure 3: The relation of effect sizes of eGFR **(A)** or serum concentrations of creatinine **(B)** to minor allele frequencies of identified SNPs. The values of differences in eGFR **(A)** or in serum concentrations of creatinine **(B)** among genotypes are shown on the *y*-axis with respect to the minor allele frequency of the corresponding SNP on the *x*-axis. SNPs identified in the EWASs are indicated.

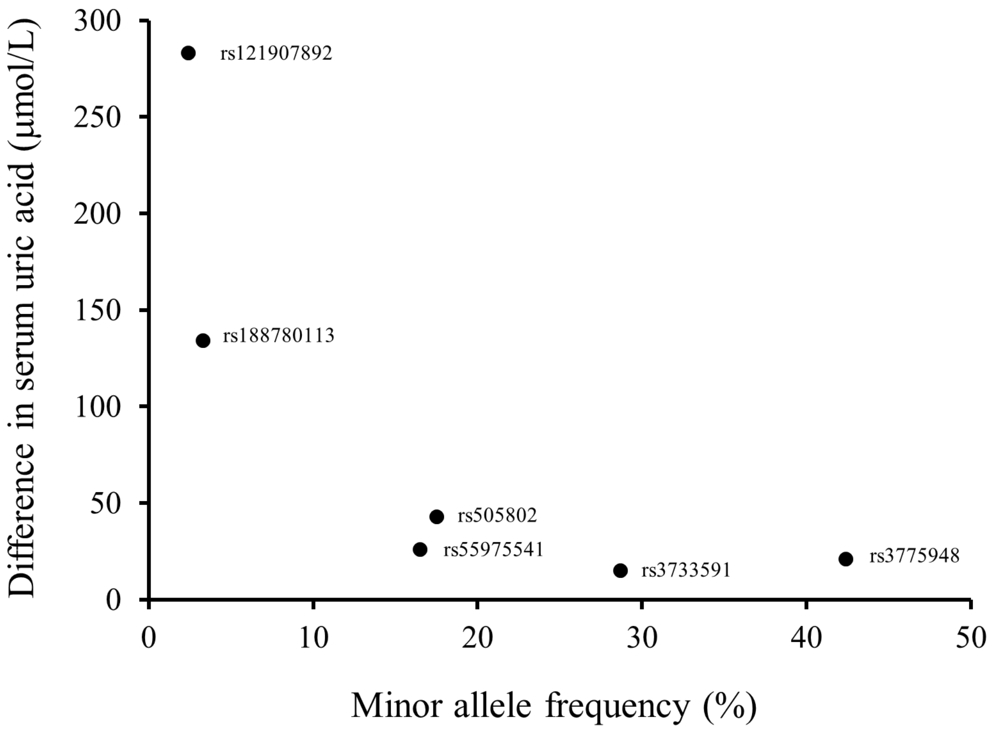

Supplementary Figure 4: The relation of effect sizes of serum concentrations of uric acid to minor allele frequencies of identified SNPs. The values of differences in serum concentrations of uric acid among genotypes are shown on the *y*-axis with respect to the minor allele frequency of the corresponding SNP on the *x*-axis. SNPs identified in the EWASs are indicated.

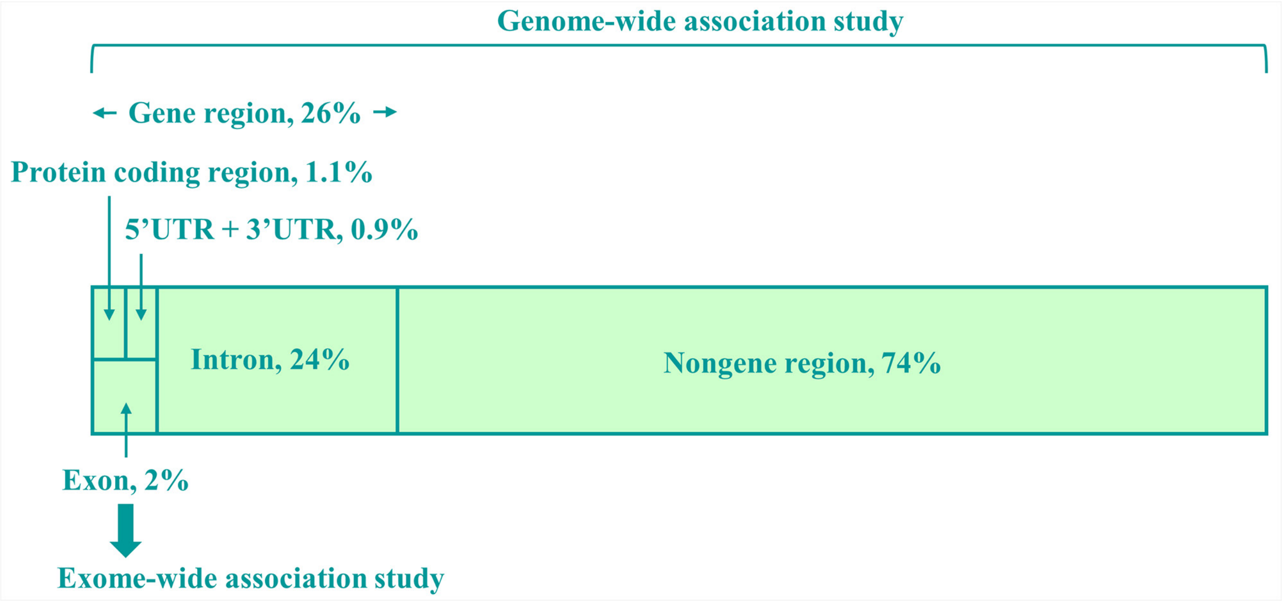

Supplementary Figure 5: The genome-wide association study (GWAS) makes use of high-throughput genotyping technologies that include up to 4.5 million markers for SNPs and copy number variations to examine their relation to clinical conditions or measurable traits. Exome array contains ~244,000 SNPs including common, low frequency, and rare variants located at whole exons. The exome-wide association study is a kind of focus genotyping method and differs from the GWAS.

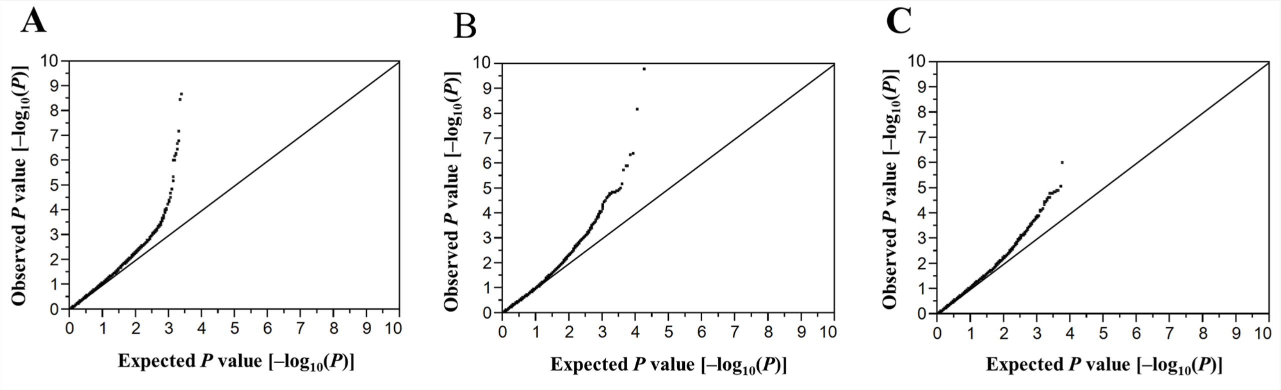

Supplementary Figure 6: Quantile-quantile plots for *P* values of genotypes in the EWASs of eGFR **(A)** or the serum concentrations of creatinine **(B)** or uric acid **(C)**. The observed *P* values (*y*-axis) are compared with the expected *P* values (*x*-axis) under the null hypothesis, with the values being plotted as $-\log_{10}(P)$.

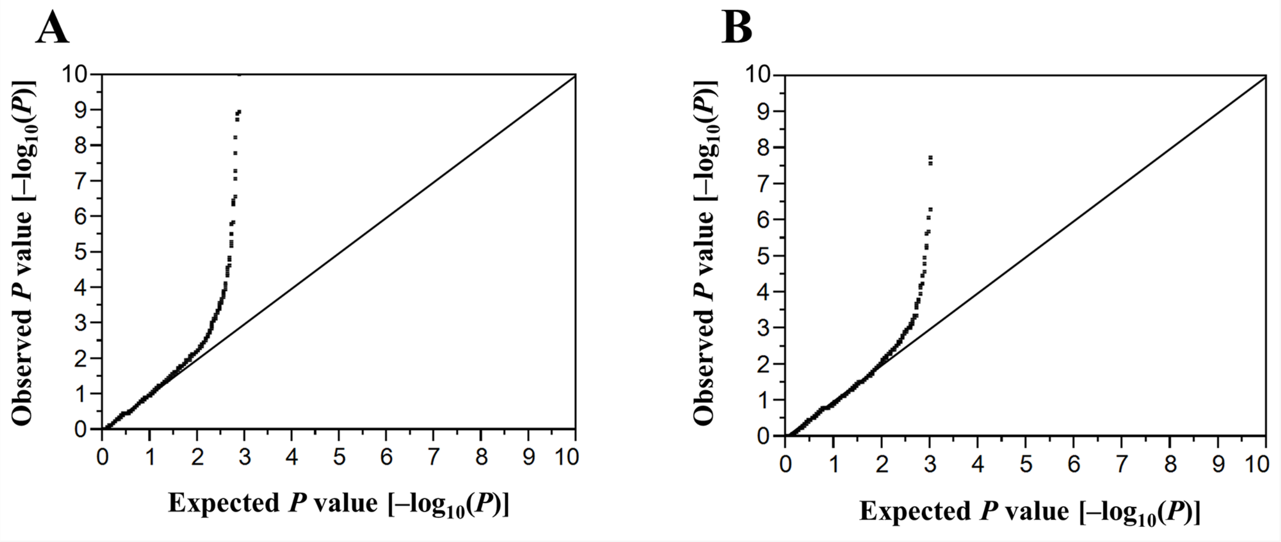

Supplementary Figure 7: Quantile-quantile plots for *P* values of allele frequencies in the EWASs of CKD **(A)** or hyperuricemia **(B)**. The observed *P* values (*y*-axis) are compared with the expected *P* values (*x*-axis) under the null hypothesis, with the values being plotted as $-\log_{10}(P)$.

Supplementary Table 1: The 49 SNPs significantly ($P < 1.21 \times 10^{-6}$) associated with CKD in the EWAS.

See Supplementary File 1

Supplementary Table 2: Genotype distributions for SNPs significantly ($P < 1.2 \times 10^{-6}$) associated with CKD in the EWAS.

See Supplementary File 2

Supplementary Table 3: Relation of the 49 SNPs identified in the EWAS to CKD as determined by multivariable logistic regression analysis.

See Supplementary File 3

Supplementary Table 4: The 35 SNPs significantly ($P < 1.21 \times 10^{-6}$) associated with hyperuricemia in the EWAS

Gene	dbSNP	Nucleotide (amino acid) substitution[a]	Chromosome: position	MAF (%)	*P* (allele)	Allele OR
IFT140	rs11648609	C/T (R621Q)	16: 1566200	10.9	$<1.00 \times 10^{-220}$	0.96
ACOT11	rs115445569	C/T (R64Q)	1: 54619935	1.1	1.52×10^{-213}	1.51
TRIML1	rs13131525	G/A (E132K)	4: 188139952	10.1	5.06×10^{-162}	0.97
WDR66	rs58098972	A/G	12: 121921491	14.5	1.03×10^{-112}	1.01
DAW1	rs10191097	T/G	2: 227911955	29.1	4.83×10^{-98}	1.04
C16orf95	rs3748393	A/C (S26A)	16: 87317167	41.7	7.78×10^{-98}	1.03
TRIM7	rs116911833	G/A (T80M)	5: 181199104	2.0	4.05×10^{-94}	1.21
DCAF4	rs17856583	C/T (L334F)	14: 72954478	0.4	1.28×10^{-89}	1.35
OR8H3	rs61751933	C/T (T16M)	11: 56122419	18.3	1.06×10^{-66}	1.04
	rs213194	G/A	6: 33227827	0.9	1.92×10^{-64}	0.75
SLC26A8	rs116528901	T/C (I393V)	6: 35961069	0.2	1.54×10^{-44}	0.97
LIPT2	rs586088	A/T (T190S)	11: 74492263	31.5	1.72×10^{-43}	0.98
CMTR2	rs144187091	T/C (I523V)	16: 71284354	1.4	1.12×10^{-39}	0.87
UBE2L3	rs5754217	G/T	22: 21585386	47.2	2.84×10^{-39}	0.97
NDRG2	rs1263872	C/G (P103A)	14: 21043299	8.1	3.19×10^{-37}	0.94
PRR16	rs17853861	C/A (P110T)	5: 120686122	6.9	1.26×10^{-36}	0.99
NUPL2	rs199844379	A/G (Y174C)	7: 23195914	0.1	6.80×10^{-36}	1.63
GATA2	rs78245253	G/C (A250P)	3: 128485850	4.6	6.69×10^{-34}	1.00
ACSF2	rs202105387	A/C (Q207P)	17: 50462542	0.2	2.57×10^{-31}	1.62
KIAA1324	rs1052878	C/T (P922L)	1: 109202996	4.9	9.99×10^{-31}	1.04
	rs11624336	G/A	14: 96727175	14.9	6.24×10^{-30}	1.01
LOC100996813	rs2453589	G/A	17: 19585538	26.0	1.34×10^{-27}	1.01
	rs6892901	C/A	5: 99359145	43.5	6.57×10^{-25}	1.06
NOTCH2	rs60854092	T/A (F1689I)	1: 119922384	4.6	5.33×10^{-24}	0.85
DAGLB	rs138713047	G/A (R646W)	7: 6409920	0.1	3.92×10^{-21}	0.34
ERICH1	rs3735933	G/A	8: 643041	46.5	5.29×10^{-20}	0.98
CCHCR1	rs1265110	G/A	6: 31151645	30.2	1.44×10^{-16}	0.97
	rs17291045	C/T	4: 160585745	4.2	3.92×10^{-14}	1.06
IL17RC	rs75116348	G/A (S56N)	3: 9917482	4.3	6.40×10^{-13}	0.99
	rs3118905	G/A	13: 50531198	1.5	3.20×10^{-11}	1.09
QRICH2	rs73996306	G/A (A69V)	17: 76304416	8.4	1.81×10^{-8}	0.92
TRIM39-RPP21	rs6931763	A/C	6: 30344155	5.4	2.69×10^{-8}	1.01
SLC9A4	rs79378995	T/C (L17P)	2: 102473809	5.1	5.57×10^{-7}	0.88
PPFIA1	rs546502	G/A (V71I)	11: 70272383	15.9	8.65×10^{-7}	0.99
STXBP2	rs188212047	G/T (L212F)	19: 7642058	0.8	8.72×10^{-7}	1.15

Allele frequencies of SNPs were analyzed with Fisher's exact test. [a]Major allele/minor allele.

Supplementary Table 5: Genotype distributions for SNPs significantly ($P < 1.2 \times 10^{-6}$) associated with hyperuricemia in the EWAS.

See Supplementary File 4

Supplementary Table 6: Relation of the 35 SNPs identified in the EWAS to hyperuricemia as determined by multivariable logistic regression analysis.

See Supplementary File 5

Supplementary Table 7: Relation of genes, chromosomal loci, and SNPs associated with eGFR, the serum creatinine concentration, or CKD in the present study to phenotypes examined in previous GWASs.

See Supplementary File 6

Supplementary Table 8: Relation of genes and SNPs associated with the serum uric acid concentration or hyperuricemia in the present study to phenotypes examined in previous GWASs

Gene	SNP	Nucleotide (amino acid) substitution	Previously examined phenotypes
Related to serum uric acid			
SLC22A12	rs121907892<br>rs505802	G/A (W258*)<br>G/A	**Serum uric acid levels** (PMID: 26902266, PMID: 19503597), **urate levels in lean, obese, and overweight individuals** (PMID: 25811787), systemic lupus erythematosus (PMID: 26606652)
ATG2A	rs188780113	G/A (R478C)	Post-bronchodilator FEV1/FVC ratio (PMID: 26634245)
CDC42BPG	rs55975541	G/A (R1237W)	**Urate levels** (PMID: 21768215), economic and political preferences (PMID: 22566634)
SLC2A9	rs3775948<br>rs3733591	G/C<br>T/C (H265R)	**Serum uric acid** (PMID: 26902266, PMID: 21294900), **gout** (PMID: 25967671, PMID: 25646370), **urate levels in lean and obese individuals** (PMID: 25811787), blood metabolite levels (PMID: 25898920), renal function–related trait (PMID: 22797727)
Related to hyperuricemia			
ACOT11	rs115445569	C/T (R64Q)	Food allergy (PMID: 25710614), non–substance-related behavioral disinhibition (PMID: 23942779)
TRIM7	rs116911833	G/A (T80M)	None
NOTCH2	rs60854092	T/A (F1689I)	Crohn's disease (PMID: 23128233), type 2 diabetes (PMID: 18372903)

Data were obtained from GWAS Catalog (http://www.ebi.ac.uk/gwas) or GWAS Central (http://www.gwascentral.org/browser). Phenotypes related to serum uric acid are shown in bold.
