# Supplementary material for: Identification of C21orf59 and ATG2A as novel determinants of renal function-related traits in Japanese by exome-wide association studies
Source: Oncotarget. 2017 Mar 30;8(28):45259–73. doi: 10.18632/oncotarget.16696 (PMC5542184; doi:10.18632/oncotarget.16696)
Supplement: Supplementary file 2 [file oncotarget-08-45259-s002.doc]

**Supplementary Table 1.** The 49 SNPs significantly (*P* < 1.21 × 10^–6^) associated with CKD in the EWAS.

________________________________________________________________________________

Gene dbSNP Nucleotide Chromosome: MAF *P* (allele) Allele

(amino acid) position (%) OR

substitution^a^

________________________________________________________________________________

*NRXN3* rs11629205 G/A 14: 78690442 33.3 1.08 × 10^–93^ 0.99

*ATG2B* rs79425071 T/C (N1089S) 14: 96316628 3.3 8.78 × 10^–82^ 1.05

rs1871686 A/G 8: 108559316 45.7 1.53 × 10^–72^ 0.97

*NAALADL1* rs141580617 G/A 11: 65054655 0.8 1.49 × 10^–65^ 1.14

*FCGRT* rs111846329 T/A (S297T) 19: 49525474 2.2 5.65 × 10^–57^ 0.77

*DLEU7* rs2812234 A/G 13: 50713678 49.3 7.93 × 10^–45^ 1.01

rs10514718 C/G 3: 61428140 11.8 2.08 × 10^–37^ 1.02

*PSMD9* rs14259 A/G (E92G) 12: 121915890 46.3 1.16 × 10^–34^ 1.08

*AUTS2* rs41510047 A/G 7: 70402415 19.5 3.50 × 10^–33^ 0.98

*SLC22A3* rs7758229 G/T 6: 160419220 24.1 5.70 × 10^–29^ 0.99

*ZNRD1ASP* rs3129012 C/T 6: 30020865 0.4 1.58 × 10^–28^ 1.93

*TET1* rs2298117 C/T 10: 68586983 43.8 1.66 × 10^–26^ 0.93

*SCNN1B* rs238551 A/G 16: 23364600 44.8 6.46 × 10^–26^ 1.02

*POTEC* rs9807633 T/G (H30P) 18: 14543058 45.1 5.70 × 10^–25^ 1.04

rs3135365 T/G 6: 32421478 18.9 2.69 × 10^–24^ 0.91

*VARS* rs707926 G/A 6: 31781043 25.6 5.53 × 10^–24^ 1.09

rs1397364 G/A 8: 11807740 32.9 1.09 × 10^–20^ 1.02

*PCNT* rs202170105 C/T (H3276Y) 21: 46443935 0.1 1.30 × 10^–20^ 1.59

*FAM73A* rs72685317 G/T 1: 77840174 10.3 4.88 × 10^–19^ 1.00

*SUPT6H* rs190479497 A/G (N800S) 17: 28684873 0.9 5.95 × 10^–18^ 0.94

PM20D1 rs117326234 G/A (T377M) 1: 205832753 0.1 8.92 × 10^–18^ 0.75

*COL18A1* rs199910738 C/T 21: 45492713 0.1 1.87 × 10^–17^ 1.45

*ANP32D* rs7956679 C/A (F46L) 12: 48472802 39.9 2.66 × 10^–17^ 0.93

*CTIF* rs2277712 C/T (P82L) 18: 48636678 14.2 3.75 × 10^–17^ 0.97

*C21orf59* rs76974938 C/T (D67N) 21: 32609946 2.4 4.47 × 10^–17^ 4.52

*WDR27* rs41265385 C/T (G218S) 6: 169667990 27.9 1.73 × 10^–16^ 1.07

*APBA2* rs7170343 C/A 15: 28984675 43.4 1.10 × 10^–14^ 0.95

*MCF2L2* rs3732602 A/G (F589S) 3: 183289130 2.2 1.20 × 10^–14^ 1.23

*LRP5* rs80358317 A/G (N1121D) 11: 68425226 0.3 6.99 × 10^–14^ 1.16

*HDAC10* rs112311672 G/A (T398M) 22: 50247974 0.3 7.25 × 10^–14^ 2.20

*FAM234B* rs192853755 C/G (I622M) 12: 13080627 0.1 6.66 × 10^–13^ 1.62

*PCLO* rs141529596 A/G (L4267S) 7: 82915186 2.3 1.62 × 10^–12^ 1.23

*MYPN* rs10823148 C/G (F628L) 10: 68166577 27.8 2.88 × 10^–12^ 0.99

*LMNA* rs4641 C/T 1: 156137743 21.8 8.41 × 10^–12^ 1.11

rs2535324 G/T 6: 30750258 47.5 1.05 × 10^–11^ 1.00

*ACAD11* rs41272317 C/A 3: 132618633 3.1 1.03 × 10^–10^ 0.89

rs209474 A/G 6: 32956807 45.1 1.16 × 10^–9^ 1.02

*IGSF10* rs138084379 A/G (I1536T) 3: 151445374 1.2 1.29 × 10^–9^ 1.43

rs490592 G/T 11: 116427650 3.0 1.36 × 10^–9^ 0.96

*CFAP206* rs139574881 G/A (R550H) 6: 87464030 0.4 1.80 × 10^–9^ 0.96

*NCR2* rs2273961 T/A (I218K) 6: 41350686 33.5 1.98 × 10^–9^ 0.96

*FCAR* rs11666735 G/A (D113N) 19: 54885501 3.2 1.99 × 10^–9^ 0.94

*NLRP12* rs146368839 C/T (A929T) 19: 53798385 1.4 5.93 × 10^–9^ 0.90

rs2442719 G/A 6: 31352761 31.3 1.60 × 10^–8^ 0.93

*DHRS7C* rs143366707 C/A (D144Y) 17: 9779876 0.6 5.29 × 10^–8^ 1.26

*RABGEF1* rs13227951 C/T 7: 66748121 8.6 9.06 × 10^–8^ 1.07

*RIF1* rs151330826 A/G (T409A) 2: 151436856 0.3 2.96 × 10^–7^ 1.27

*DCBLD2* rs9838238 T/C (I144M) 3: 98881541 1.8 3.45 × 10^–7^ 1.01

rs1233397 C/T 6: 29577938 47.6 4.81 × 10^–7^ 1.05

________________________________________________________________________________

Allele frequencies of SNPs were analyzed with Fisher’s exact test. ^a^Major allele/minor allele. OR, odds ratio.
