# Supplementary material for: Identification of C21orf59 and ATG2A as novel determinants of renal function-related traits in Japanese by exome-wide association studies
Source: Oncotarget. 2017 Mar 30;8(28):45259–73. doi: 10.18632/oncotarget.16696 (PMC5542184; doi:10.18632/oncotarget.16696)
Supplement: Supplementary file 3 [file oncotarget-08-45259-s003.doc]

**Supplementary Table 2.** Genotype distributions for SNPs significantly (*P* < 1.2 × 10^–6^) associated with CKD in the EWAS.

____________________________________________________________________________________________________________

SNP CKD H-W *P* Controls H-W *P*

____________________________________________________________________________________________________________

rs11629205 G/A *GG* *GA* *AA* *GG* *GA* *AA*

1437 (43.96) 1484 (45.40) 348 (10.64) 0.2378 846 (44.74) 824 (43.57) 221 (11.69) 0.3527

rs79425071 T/C (N1089S) *TT* *TC* *CC*  *TT* *TC* *CC*

3060 (93.81) 197 (6.04) 5 (0.15) 0.3808 1778 (94.18) 106 (5.61) 4 (0.21) 0.0881

rs1871686 A/G *AA* *AG* *GG*  *AA* *AG* *GG*

983 (30.06) 1609 (49.21) 678 (20.73) 0.6722 553 (29.25) 931 (49.23) 407 (21.52) 0.6773

rs141580617 G/A *GG* *GA* *AA* *GG* *GA* *AA*

3220 (98.47) 49 (1.50) 1 (0.03) 0.1784 1866 (98.68) 24 (1.27) 1 (0.05) 0.0829

rs111846329 T/A (S297T) *TT* *TA* *AA*  *TT* *TA* *AA*

3133 (95.81) 134 (4.10) 3 (0.09) 0.1853 1789 (94.61) 100 (5.29) 2 (0.10) 0.6508

rs2812234 A/G *AA* *AG* *GG*  *AA* *AG* *GG*

877 (26.83) 1562 (47.78) 830 (25.39) 0.0118 508 (26.86) 907 (47.97) 476 (25.17) 0.0804

rs10514718 C/G *CC* *CG* *GG*  *CC* *CG* *GG*

2550 (77.98) 666 (20.37) 54 (1.65) 0.1793 1482 (78.37) 378 (19.99) 31 (1.64) 0.2190

rs14259 A/G (E92G) *AA* *AG* *GG*  *AA* *AG* *GG*

872 (26.71) 1676 (51.33) 717 (21.96) 0.1065 540 (28.57) 968 (51.22) 382 (20.21) 0.1792

rs41510047 A/G *AA* *AG* *GG*  *AA* *AG* *GG*

2118 (64.77) 1042 (31.87) 110 (3.36) 0.1968 1239 (65.52) 563 (29.77) 89 (4.71) 0.0193

rs7758229 G/T *GG* *GT* *TT*  *GG* *GT* *TT*

1927 (58.95) 1152 (35.24) 190 (5.81) 0.3061 1091 (57.76) 703 (37.21) 95 (5.03) 0.2022

rs3129012 C/T *CC* *CT* *TT*  *CC* *CT* *TT*

3240 (99.08) 30 (0.92) 0 (0) 1.0000 1882 (99.52) 9 (0.48) 0 (0) 1.0000

rs2298117 C/T *CC* *CT* *TT*  *CC* *CT* *TT*

1039 (31.77) 1602 (48.99) 629 (19.24) 0.8036 565 (29.88) 934 (49.39) 392 (20.73) 0.8893

rs238551 A/G *AA* *AG* *GG*  *AA* *AG* *GG*

1010 (30.90) 1634 (49.98) 625 (19.12) 0.4563 611 (32.31) 909 (48.07) 371 (19.62) 0.3263

rs9807633 T/G (H30P) *TT* *TG* *GG*  *TT* *TG* *GG*

954 (29.29) 1609 (49.40) 694 (21.31) 0.7510 561 (29.81) 951 (50.53) 370 (19.66) 0.3762

rs3135365 T/G *TT* *TG* *GG*  *TT* *TG* *GG*

2198 (67.26) 964 (29.50) 106 (3.24) 1.0000 1219 (64.46) 607 (32.10) 65 (3.44) 0.3412

rs707926 G/A *GG* *GA* *AA* *GG* *GA* *AA*

1792 (54.80) 1238 (37.86) 240 (7.34) 0.2061 1055 (55.79) 740 (39.13) 96 (5.08) 0.0218

rs1397364 G/A *GG* *GA* *AA* *GG* *GA* *AA*

1509 (46.18) 1387 (42.44) 372 (11.38) 0.0511 869 (45.96) 826 (43.68) 196 (10.36) 1.0000

rs202170105 C/T (H3276Y) *CC* *CT* *TT*  *CC* *CT* *TT*

3259 (99.66) 11 (0.34) 0 (0) 1.0000 1887 (99.79) 4 (0.21) 0 (0) 1.0000

rs72685317 G/T *GG* *GT* *TT*  *GG* *GT* *TT*

2644 (80.86) 592 (18.10) 34 (1.04) 0.8475 1531 (80.96) 337 (17.82) 23 (1.22) 0.3751

rs190479497 A/G (N800S) *AA* *AG* *GG*  *AA* *AG* *GG*

3200 (97.86) 70 (2.14) 0 (0) 1.0000 1848 (97.73) 43 (2.27) 0 (0) 1.0000

rs117326234 G/A (T377M) *GG* *GA* *AA* *GG* *GA* *AA*

3257 (99.60) 13 (0.40) 0 (0) 1.0000 1881 (99.47) 10 (0.53) 0 (0) 1.0000

rs199910738 C/T *CC* *CT* *TT*  *CC* *CT* *TT*

3260 (99.69) 10 (0.31) 0 (0) 1.0000 1887 (99.79) 4 (0.21) 0 (0) 1.0000

rs7956679 C/A (F46L) *CC* *CA* *AA*  *CC* *CA* *AA*

1118 (35.16) 1576 (49.56) 486 (15.28) 0.0763 639 (33.93) 913 (48.49) 331 (17.58) 0.8870

rs2277712 C/T (P82L) *CC* *CT* *TT*  *CC* *CT* *TT*

2406 (73.58) 785 (24.01) 79 (2.41) 0.1191 1370 (72.45) 484 (25.59) 37 (1.96) 0.5218

rs76974938 C/T (D67N) *CC* *CT* *TT*  *CC* *CT* *TT*

2341 (93.45) 164 (6.55) 0 (0) 0.1116 1791 (98.52) 27 (1.48) 0 (0) 1.0000

rs41265385 C/T (G218S) *CC* *CT* *TT*  *CC* *CT* *TT*

1682 (51.45) 1326 (40.56) 261 (7.99) 1.0000 1011 (53.47) 740 (39.13) 140 (7.40) 0.7705

rs7170343 C/A *CC* *CA* *AA*  *CC* *CA* *AA*

999 (34.49) 1353 (46.70) 545 (18.81) 0.0223 606 (32.05) 932 (49.28) 353 (18.67) 0.8883

rs3732602 A/G (F589S) *AA* *AG* *GG*  *AA* *AG* *GG*

3104 (94.93) 159 (4.86) 7 (0.21) 0.0070 1810 (95.72) 80 (4.23) 1 (0.05) 0.5924

rs80358317 A/G (N1121D) *AA* *AG* *GG*  *AA* *AG* *GG*

3250 (99.39) 20 (0.61) 0 (0) 1.0000 1881 (99.47) 10 (0.53) 0 (0) 1.0000

rs112311672 G/A (T398M) *GG* *GA* *AA* *GG* *GA* *AA*

3251 (99.42) 19 (0.58) 0 (0) 1.0000 1886 (99.74) 5 (0.26) 0 (0) 1.0000

rs192853755 C/G (I622M) *CC* *CG* *GG*  *CC* *CG* *GG*

3256 (99.57) 14 (0.43) 0 (0) 1.0000 1886 (99.74) 5 (0.26) 0 (0) 1.0000

rs141529596 A/G (L4267S) *AA* *AG* *GG*  *AA* *AG* *GG*

3092 (94.56) 178 (5.44) 0 (0) 0.1755 1808 (95.61) 82 (4.34) 1 (0.05) 0.6104

rs10823148 C/G (F628L) *CC* *CG* *GG*  *CC* *CG* *GG*

1743 (53.35) 1276 (39.06) 248 (7.59) 0.5067 998 (52.78) 752 (39.77) 141 (7.45) 1.0000

rs4641 C/T *CC* *CT* *TT*  *CC* *CT* *TT*

1940 (59.33) 1145 (35.01) 185 (5.66) 0.3507 1176 (62.19) 622 (32.89) 93 (4.92) 0.3734

rs2535324 G/T *GG* *GT* *TT*  *GG* *GT* *TT*

884 (27.04) 1628 (49.80) 757 (23.16) 0.8886 537 (28.40) 894 (47.28) 460 (24.32) 0.0213

rs41272317 C/A *CC* *CA* *AA*  *CC* *CA* *AA*

3081 (94.22) 187 (5.72) 2 (0.06) 1.0000 1767 (93.44) 124 (6.56) 0 (0) 0.2641

rs209474 A/G *AA* *AG* *GG*  *AA* *AG* *GG*

978 (29.93) 1633 (49.97) 657 (20.10) 0.6209 570 (30.14) 956 (50.56) 365 (19.30) 0.3286

rs138084379 A/G (I1536T) *AA* *AG* *GG*  *AA* *AG* *GG*

3179 (97.22) 91 (2.78) 0 (0) 1.0000 1854 (98.04) 37 (1.96) 0 (0) 1.0000

rs490592 G/T *GG* *GT* *TT*  *GG* *GT* *TT*

3086 (94.37) 181 (5.54) 3 (0.09) 0.7483 1781 (94.18) 108 (5.71) 2 (0.11) 0.6790

rs139574881 G/A (R550H) *GG* *GA* *AA* *GG* *GA* *AA*

3239 (99.08) 30 (0.92) 0 (0) 1.0000 1873 (99.05) 18 (0.95) 0 (0) 1.0000

rs2273961 T/A (I218K) *TT* *TA* *AA*  *TT* *TA* *AA*

1457 (45.03) 1413 (43.66) 366 (11.31) 0.4049 803 (42.46) 886 (46.86) 202 (10.68) 0.0733

rs11666735 G/A (D113N) *GG* *GA* *AA* *GG* *GA* *AA*

3071 (93.92) 197 (6.02) 2 (0.06) 0.7693 1769 (93.55) 120 (6.35) 2 (0.10) 1.0000

rs146368839 C/T (A929T) *CC* *CT* *TT*  *CC* *CT* *TT*

3183 (97.34) 87 (2.66) 0 (0) 1.0000 1835 (97.04) 56 (2.96) 0 (0) 1.0000

rs2442719 G/A *GG* *GA* *AA* *GG* *GA* *AA*

1573 (48.10) 1404 (42.94) 293 (8.96) 0.4328 864 (45.69) 847 (44.79) 180 (9.52) 0.2040

rs143366707 C/A (D144Y) *CC* *CA* *AA*  *CC* *CA* *AA*

3246 (99.27) 24 (0.73) 0 (0) 1.0000 1880 (99.42) 11 (0.58) 0 (0) 1.0000

rs13227951 C/T *CC* *CT* *TT*  *CC* *CT* *TT*

2705 (82.72) 543 (16.61) 22 (0.67) 0.3929 1582 (83.66) 298 (15.76) 11 (0.58) 0.5525

rs151330826 A/G (T409A) *AA* *AG* *GG*  *AA* *AG* *GG*

3248 (99.33) 22 (0.67) 0 (0) 1.0000 1881 (99.47) 10 (0.53) 0 (0) 1.0000

rs9838238 T/C (I144M) *TT* *TC* *CC*  *TT* *TC* *CC*

3154 (96.45) 115 (3.52) 1 (0.03) 1.0000 1824 (96.46) 67 (3.54) 0 (0) 1.0000

rs1233397 C/T *CC* *CT* *TT*  *CC* *CT* *TT*

891 (27.25) 1579 (48.29) 800 (24.46) 0.0587 536 (28.35) 917 (48.49) 438 (23.16) 0.2308

____________________________________________________________________________________________________________

Data and values in parentheses are numbers of subjects and percentages, respectively. H-W *P*, *P* value for Hardy-Weinberg equilibrium.
