# Supplementary material for: Identification of C21orf59 and ATG2A as novel determinants of renal function-related traits in Japanese by exome-wide association studies
Source: Oncotarget. 2017 Mar 30;8(28):45259–73. doi: 10.18632/oncotarget.16696 (PMC5542184; doi:10.18632/oncotarget.16696)
Supplement: Supplementary file 4 [file oncotarget-08-45259-s004.doc]

**Supplementary Table 3.** Relation of the 49 SNPs identified in the EWAS to CKD as determined by multivariable logistic regression analysis.

__________________________________________________________________________________________________________________________

SNP Dominant Recessive Additive 1 Additive 2

____________________ ____________________ ____________________ ____________________

*P* OR (95% CI) *P* OR (95% CI) *P* OR (95% CI) *P* OR (95% CI)

__________________________________________________________________________________________________________________________

rs11629205 G/A 0.7673 0.0967 0.3808 0.1964

rs79425071 T/C (N1089S) 0.9613 0.6194 0.9736 0.6197

rs1871686 A/G 0.5185 0.6973 0.4081 0.9308

rs141580617 G/A 0.6196 0.7533 0.5897 0.7540

rs111846329 T/A (S297T) 0.9893 0.6191 0.9630 0.6193

rs2812234 A/G 0.7433 0.9032 0.6918 0.9056

rs10514718 C/G 0.6885 0.4303 0.5334 0.4559

rs14259 A/G (E92G) 0.2267 0.3369 0.3426 0.1870

rs41510047 A/G 0.8573 0.0558 0.4625 0.0725

rs7758229 G/T 0.2140 0.6739 0.1560 0.8723

rs3129012 C/T 0.1915 ND 0.1915 ND

rs2298117 C/T 0.2718 0.1138 0.5454 0.0941

rs238551 A/G 0.2828 0.9867 0.2506 0.5932

rs9807633 T/G (H30P) 0.1698 0.5917 0.2052 0.2684

rs3135365 T/G 0.1025 0.9680 0.0894 0.8661

rs707926 G/A 0.4239 0.0042 1.54 (1.14–2.08) 0.9753 0.0052 1.53 (1.14–2.09)

rs1397364 G/A 0.8231 0.2584 0.8787 0.3025

rs202170105 C/T (H3276Y) 0.9112 ND 0.9112 ND

rs72685317 G/T 0.7363 0.8937 0.7529 0.8814

rs190479497 A/G (N800S) 0.8714 ND 0.8714 ND

rs117326234 G/A (T377M) 0.8212 ND 0.8212 ND

rs199910738 C/T 0.3244 ND 0.3244 ND

rs7956679 C/A (F46L) 0.5072 0.6929 0.5720 0.5468

rs2277712 C/T (P82L) 0.5613 0.8559 0.5804 0.8178

rs76974938 C/T (D67N) 0.0420 1.52 (1.00–2.42) ND 0.0420 1.52 (1.00–2.42) ND

rs41265385 C/T (G218S) 0.0931 0.7123 0.1000 0.4586

rs7170343 C/A 0.7980 0.7586 0.7013 0.9232

rs3732602 A/G (F589S) 0.2052 0.4119 0.2529 0.4069

rs80358317 A/G (N1121D) 0.7178 ND 0.7178 ND

rs112311672 G/A (T398M) 0.0492 3.33 (1.00–13.35) ND 0.0492 3.33 (1.00–13.35) ND

rs192853755 C/G (I622M) 0.1067 ND 0.1067 ND

rs141529596 A/G (L4267S) 0.1896 0.2522 0.1634 0.2539

rs10823148 C/G (F628L) 0.7389 0.6393 0.6178 0.7317

rs4641 C/T 0.0577 0.4954 0.0758 0.3305

rs2535324 G/T 0.2852 0.6804 0.1963 0.7186

rs41272317 C/A 0.2694 0.0220 >100 (ND) 0.2094 0.0224 >100 (ND)

rs209474 A/G 0.4292 0.5175 0.2926 0.9713

rs138084379 A/G (I1536T) 0.1140 ND 0.1140 ND

rs490592 G/T 0.7074 0.5933 0.6498 0.5960

rs139574881 G/A (R550H) 0.6407 ND 0.6407 ND

rs2273961 T/A (I218K) 0.3528 0.1845 0.1564 0.4201

rs11666735 G/A (D113N) 0.5868 0.9848 0.5837 0.9879

rs146368839 C/T (A929T) 0.5451 ND 0.5451 ND

rs2442719 G/A 0.1526 0.3081 0.2382 0.1900

rs143366707 C/A (D144Y) 0.5472 ND 0.5472 ND

rs13227951 C/T 0.5198 0.2438 0.6588 0.2375

rs151330826 A/G (T409A) 0.5010 ND 0.5010 ND

rs9838238 T/C (I144M) 0.4299 0.3018 0.4738 0.3007

rs1233397 C/T 0.3158 0.2186 0.5404 0.1721

__________________________________________________________________________________________________________________________

Multivariable logistic regression analysis was performed with adjustment for age, sex, and the prevalence of hypertension and diabetes mellitus. Based on Bonferroni’s correction, a *P* value of <2.55 × 10^–4^ (0.05/196) was considered statistically significant. OR, odds ratio; CI, confidence interval; ND, not determined.
