# Supplementary material for: Identification of C21orf59 and ATG2A as novel determinants of renal function-related traits in Japanese by exome-wide association studies
Source: Oncotarget. 2017 Mar 30;8(28):45259–73. doi: 10.18632/oncotarget.16696 (PMC5542184; doi:10.18632/oncotarget.16696)
Supplement: Supplementary file 5 [file oncotarget-08-45259-s005.doc]

**Supplementary Table 5.** Genotype distributions for SNPs significantly (*P* < 1.2 × 10^–6^) associated with hyperuricemia in the EWAS.

_____________________________________________________________________________________________________________________

SNP Hyperuricemia H-W *P* Controls H-W *P*

_____________________________________________________________________________________________________________________

rs11648609 C/T (R621Q) *CC* *CT* *TT* *CC* *CT* *TT*

1639 (80.15) 380 (18.58) 26 (1.27) 0.4814 7652 (79.37) 1856 (19.25) 133 (1.38) 0.0959

rs115445569 C/T (R64Q) *CC* *CT* *TT* *CC* *CT* *TT*

1983 (96.97) 61 (2.98) 1 (0.05) 0.3843 9442 (97.97) 194 (2.01) 2 (0.02) 0.2686

rs13131525 G/A (E132K) *GG* *GA* *AA*  *GG* *GA* *AA*

1668 (81.56) 348 (17.02) 29 (1.42) 0.0347 7788 (80.77) 1743 (18.08) 111 (1.15) 0.2211

rs58098972 A/G *AA* *AG* *GG*  *AA* *AG* *GG*

1515 (74.08) 472 (23.08) 58 (2.84) 0.0068 7089 (73.53) 2344 (24.31) 208 (2.16) 0.3833

rs10191097 T/G *TT* *TG* *GG*  *TT* *TG* *GG*

1002 (49.02) 858 (41.98) 184 (9.00) 1.0000 4844 (50.24) 3987 (41.36) 810 (8.40) 0.8050

rs3748393 A/C (S26A) *AA* *AC* *CC*  *AA* *AC* *CC*

692 (33.84) 1000 (48.90) 353 (17.26) 0.8202 3422 (35.50) 4547 (47.17) 1671 (17.33) 0.0163

rs116911833 G/A (T80M) *GG* *GA* *AA*  *GG* *GA* *AA*

1974 (96.53) 68 (3.32) 3 (0.15) 0.0268 9255 (96.00) 383 (3.97) 3 (0.03) 1.0000

rs17856583 C/T (L334F) *CC* *CT* *TT* *CC* *CT* *TT*

2025 (99.02) 20 (0.98) 0 (0) 1.0000 9571 (99.27) 70 (0.73) 0 (0) 1.0000

rs61751933 C/T (T16M) *CC* *CT* *TT* *CC* *CT* *TT*

1458 (71.72) 522 (25.67) 53 (2.61) 0.4446 6990 (72.59) 2403 (24.96) 236 (2.45) 0.0918

rs213194 G/A *GG* *GA* *AA*  *GG* *GA* *AA*

2013 (98.44) 32 (1.56) 0 (0) 1.0000 9440 (97.92) 201 (2.08) 0 (0) 0.6288

rs116528901 T/C (I393V) *TT* *TC* *CC*  *TT* *TC* *CC*

2038 (99.66) 7 (0.34) 0 (0) 1.0000 9607 (99.65) 34 (0.35) 0 (0) 1.0000

rs586088 A/T (T190S) *AA* *AT* *TT*  *AA* *AT* *TT*

969 (47.43) 865 (42.34) 209 (10.23) 0.4412 4483 (46.50) 4173 (43.29) 984 (10.21) 0.7783

rs144187091 T/C (I523V) *TT* *TC* *CC*  *TT* *TC* *CC*

1991 (97.46) 52 (2.54) 0 (0) 1.0000 9365 (97.14) 272 (2.82) 4 (0.04) 0.1449

rs5754217 G/T *GG* *GT* *TT*  *GG* *GT* *TT*

583 (28.52) 1021 (49.95) 440 (21.53) 0.8939 2705 (28.06) 4774 (49.52) 2161 (22.42) 0.5398

rs1263872 C/G (P103A) *CC* *CG* *GG*  *CC* *CG* *GG*

1740 (85.13) 294 (14.38) 10 (0.49) 0.6398 8139 (84.43) 1432 (14.85) 69 (0.72) 0.4956

rs17853861 C/A (P110T) *CC* *CA* *AA*  *CC* *CA* *AA*

1776 (86.85) 258 (12.61) 11 (0.54) 0.6015 8354 (86.65) 1238 (12.84) 49 (0.51) 0.6359

rs199844379 A/G (Y174C) *AA* *AG* *GG*  *AA* *AG* *GG*

2035 (99.51) 10 (0.49) 0 (0) 1.0000 9610 (99.70) 29 (0.30) 0 (0) 1.0000

rs78245253 G/C (A250P) *GG* *GC* *CC*  *GG* *GC* *CC*

1854 (90.66) 186 (9.10) 5 (0.24) 0.8074 8743 (90.69) 870 (9.02) 28 (0.29) 0.2186

rs202105387 A/C (Q207P) *AA* *AC* *CC*  *AA* *AC* *CC*

2033 (99.41) 12 (0.59) 0 (0) 1.0000 9606 (99.64) 35 (0.36) 0 (0) 1.0000

rs1052878 C/T (P922L) *CC* *CT* *TT* *CC* *CT* *TT*

1842 (90.08) 197 (9.63) 6 (0.29) 0.6506 8725 (90.51) 885 (9.18) 30 (0.31) 0.1537

rs11624336 G/A *GG* *GA* *AA*  *GG* *GA* *AA*

1475 (72.16) 508 (24.85) 61 (2.99) 0.0412 6940 (71.99) 2450 (25.42) 250 (2.59) 0.0592

rs2453589 G/A *GG* *GA* *AA*  *GG* *GA* *AA*

1119 (54.75) 779 (38.11) 146 (7.14) 0.5292 5291 (54.88) 3683 (38.20) 667 (6.92) 0.4588

rs6892901 C/A *CC* *CA* *AA*  *CC* *CA* *AA*

640 (31.30) 988 (48.31) 417 (20.39) 0.3248 3162 (32.80) 4660 (48.33) 1819 (18.87) 0.1640

rs60854092 T/A (F1689I) *TT* *TA* *AA*  *TT* *TA* *AA*

1876 (91.73) 166 (8.12) 3 (0.15) 1.0000 8718 (90.44) 901 (9.34) 21 (0.22) 0.7426

rs138713047 G/A (R646W) *GG* *GA* *AA*  *GG* *GA* *AA*

2043 (99.90) 2 (0.10) 0 (0) 1.0000 9613 (99.71) 28 (0.29) 0 (0) 1.0000

rs3735933 G/A *GG* *GA* *AA*  *GG* *GA* *AA*

605 (29.58) 1008 (49.29) 432 (21.13) 0.7552 2772 (28.76) 4799 (49.78) 2069 (21.46) 0.9347

rs1265110 G/A *GG* *GA* *AA*  *GG* *GA* *AA*

1015 (49.63) 854 (41.76) 176 (8.61) 0.3540 4706 (48.82) 4050 (42.02) 883 (9.16) 0.9134

rs17291045 C/T *CC* *CT* *TT*  *CC* *CT* *TT*

1872 (91.54) 166 (8.12) 7 (0.34) 0.1113 8852 (91.82) 774 (8.03) 15 (0.15) 0.7982

rs75116348 G/A (S56N) *GG* *GA* *AA*  *GG* *GA* *AA*

1882 (92.03) 160 (7.82) 3 (0.15) 1.0000 8865 (91.95) 759 (7.87) 17 (0.18) 0.7963

rs3118905 G/A *GG* *GA* *AA*  *GG* *GA* *AA*

1984 (97.06) 59 (2.89) 1 (0.05) 0.3652 9378 (97.28) 260 (2.70) 2 (0.02) 0.7018

rs73996306 G/A (A69V) *GG* *GA* *AA*  *GG* *GA* *AA*

1739 (85.04) 292 (14.28) 14 (0.68) 0.6443 8085 (83.86) 1480 (15.35) 76 (0.79) 0.3573

rs6931763 A/C *AA* *AC* *CC*  *AA* *AC* *CC*

1831 (89.54) 207 (10.12) 7 (0.34) 0.6619 8640 (89.63) 967 (10.03) 33 (0.34) 0.2694

rs79378995 T/C (L17P) *TT* *TC* *CC*  *TT* *TC* *CC*

1855 (90.71) 187 (9.14) 3 (0.15) 0.6230 8639 (89.61) 978 (10.14) 24 (0.25) 0.6125

rs546502 G/A (V71I) *GG* *GA* *AA*  *GG* *GA* *AA*

1478 (72.28) 515 (25.18) 52 (2.54) 0.3888 6930 (71.88) 2468 (25.60) 243 (2.52) 0.1951

rs188212047 G/T (L212F) *GG* *GT* *TT*  *GG* *GT* *TT*

1996 (97.99) 41 (2.01) 0 (0) 1.0000 9443 (98.24) 169 (1.76) 0 (0) 1.0000

_____________________________________________________________________________________________________________________

Data and values in parentheses are numbers of subjects and percentages, respectively. H-W *P*, *P* value for Hardy-Weinberg equilibrium.
