# Supplementary material for: Identification of C21orf59 and ATG2A as novel determinants of renal function-related traits in Japanese by exome-wide association studies
Source: Oncotarget. 2017 Mar 30;8(28):45259–73. doi: 10.18632/oncotarget.16696 (PMC5542184; doi:10.18632/oncotarget.16696)
Supplement: Supplementary file 6 [file oncotarget-08-45259-s006.doc]

**Supplementary Table 6.** Relation of the 35 SNPs identified in the EWAS to hyperuricemia as determined by multivariable logistic regression analysis.

__________________________________________________________________________________________________________________________

SNP Dominant Recessive Additive 1 Additive 2

____________________ ____________________ ____________________ ____________________

*P* OR (95% CI) *P* OR (95% CI) *P* OR (95% CI) *P* OR (95% CI)

__________________________________________________________________________________________________________________________

rs11648609 C/T (R621Q) 0.3441 0.5472 0.4073 0.5168

rs115445569 C/T (R64Q) 0.0228 1.43 (1.05–1.93) 0.5153 0.0266 1.42 (1.04–1.92) 0.5114

rs13131525 G/A (E132K) 0.1620 0.6879 0.1238 0.7508

rs58098972 A/G 0.6210 0.1344 0.3534 0.1590

rs10191097 T/G 0.1864 0.2358 0.3137 0.1601

rs3748393 A/C (S26A) 0.2847 0.8942 0.2769 0.5504

rs116911833 G/A (T80M) 0.2547 0.0331 7.21 (1.19–43.73) 0.1557 0.0337 7.15 (1.18–43.43)

rs17856583 C/T (L334F) 0.4023 ND 0.4023 ND

rs61751933 C/T (T16M) 0.2369 0.8781 0.2363 0.7921

rs213194 G/A 0.1375 ND 0.1375 ND

rs116528901 T/C (I393V) 0.8526 ND 0.8526 ND

rs586088 A/T (T190S) 0.5997 0.7510 0.5101 0.9106

rs144187091 T/C (I523V) 0.4265 0.1090 0.5260 0.1086

rs5754217 G/T 0.5360 0.4113 0.7139 0.3730

rs1263872 C/G (P103A) 0.5037 0.1442 0.6914 0.1408

rs17853861 C/A (P110T) 0.9756 0.9275 0.9892 0.9272

rs199844379 A/G (Y174C) 0.2822 ND 0.2822 ND

rs78245253 G/C (A250P) 0.7697 0.7748 0.7309 0.7791

rs202105387 A/C (Q207P) 0.1588 ND 0.1588 ND

rs1052878 C/T (P922L) 0.3814 0.8261 0.3542 0.8389

rs11624336 G/A 0.6649 0.4480 0.5071 0.4904

rs2453589 G/A 0.7115 0.5276 0.5582 0.6239

rs6892901 C/A 0.1044 0.1678 0.2198 0.0725

rs60854092 T/A (F1689I) 0.0377 0.83 (0.69–0.99) 0.4797 0.0455 0.84 (0.70–1.00) 0.4628

rs138713047 G/A (R646W) 0.0697 ND 0.0697 ND

rs3735933 G/A 0.8500 0.7469 0.9317 0.7483

rs1265110 G/A 0.9018 0.6942 0.7971 0.7550

rs17291045 C/T 0.9998 0.1611 0.8209 0.1622

rs75116348 G/A (S56N) 0.9315 0.8757 0.9149 0.8767

rs3118905 G/A 0.5201 0.3236 0.5807 0.3228

rs73996306 G/A (A69V) 0.1466 0.8703 0.1477 0.8301

rs6931763 A/C 0.9535 0.6617 0.9834 0.6621

rs79378995 T/C (L17P) 0.2009 0.3602 0.2442 0.3512

rs546502 G/A (V71I) 0.9932 0.6480 0.8970 0.6580

rs188212047 G/T (L212F) 0.5353 ND 0.5353 ND

__________________________________________________________________________________________________________________________

Multivariable logistic regression analysis was performed with adjustment for age and sex. Based on Bonferroni’s correction, a *P* value of <3.57 × 10^–4^ (0.05/140) was considered statistically significant. ND, not determined.
