# Supplementary material for: Identification of C21orf59 and ATG2A as novel determinants of renal function-related traits in Japanese by exome-wide association studies
Source: Oncotarget. 2017 Mar 30;8(28):45259–73. doi: 10.18632/oncotarget.16696 (PMC5542184; doi:10.18632/oncotarget.16696)
Supplement: Supplementary file 7 [file oncotarget-08-45259-s007.doc]

**Supplementary Table 7.** Relation of genes, chromosomal loci, and SNPs associated with eGFR, the serum creatinine concentration, or CKD in the present study to phenotypes examined in previous GWASs.

| Gene or locus | SNP | | Nucleotide  (amino acid)  substitution | Previously examined phenotypes |
| --- | --- | --- | --- | --- |
| Related to eGFR and CKD | |  | | |
| *C21orf59* | rs76974938 | | C/T (D67N) | None |
| Related to eGFR | |  | | |
| *GGCT* | rs115910467 | | C/T (R108H) | None |
| *COL6A5* | rs200982668 | | G/A (E2501K) | [Body mass index](http://www.ebi.ac.uk/gwas/search?query=Body mass index) (PMID: 24348519) |
| *MOB3C* | rs139537100 | | C/T (R24Q) | None |
| *CXCL8* | rs188378669 | | G/T (E31*) | None |
| *PLCB2* | rs200787930 | | C/T (E1095K) | [Schizophrenia](http://www.ebi.ac.uk/gwas/search?query=Schizophrenia) (PMID: 25056061, PMID: 21926974) |
| *MARCH1* | rs61734696 | | G/T (Q137K) | [Urinary uromodulin levels](http://www.ebi.ac.uk/gwas/search?query=Urinary uromodulin levels) (PMID: 24578125), [post-bronchodilator FEV1/FVC ratio](http://www.ebi.ac.uk/gwas/search?query=Post bronchodilator FEV1/FVC ratio) (PMID: 26634245), [diisocyanate-induced asthma](http://www.ebi.ac.uk/gwas/search?query=Diisocyanate-induced asthma) (PMID: 25918132), [type 2 diabetes](http://www.ebi.ac.uk/gwas/search?query=Type 2 diabetes) (PMID: 21490949) |
| *VPS33B* | rs199921354 | | C/T (R80Q) | [Type 2 diabetes](http://www.ebi.ac.uk/gwas/search?query=Type 2 diabetes) (PMID: 24509480) |
| *TMOD4* | rs115287176 | | G/A (R277W) | None |
| *TNC* | rs138406927 | | C/T (A1096T) | [Developmental language disorder](http://www.ebi.ac.uk/gwas/search?query=Developmental language disorder (syntactic complexity)) (PMID: 27016271), [post-bronchodilator FEV1 in COPD](http://www.ebi.ac.uk/gwas/search?query=Post bronchodilator FEV1 in COPD) (PMID: 26634245), [plasma omega-3 polyunsaturated fatty acid level](http://www.ebi.ac.uk/gwas/search?query=Plasma omega-3 polyunsaturated fatty acid level (eicosapentaenoic acid)) (PMID: 26584805), [cerebral amyloid deposition in APOE ε4 noncarriers](http://www.ebi.ac.uk/gwas/search?query=Cerebral amyloid deposition in APOEe4 non-carriers (PET imaging)) (PMID: 26252872), [glucose homeostasis traits](http://www.ebi.ac.uk/gwas/search?query=Glucose homeostasis traits) (PMID: 25524916) |
| *ZNF77* | rs146879198 | | G/A (R340*) | None |
| *COL6A3* | rs146092501 | | C/T (E1386K) | [Aging](http://www.ebi.ac.uk/gwas/search?query=Aging (time to event)) (PMID: 21782286), [prostate cancer](http://www.ebi.ac.uk/gwas/search?query=Prostate cancer) (PMID: 21743467) |
| *ADGRL3* | rs192210727 | | G/T (R580I) | [Post-bronchodilator FEV1/FVC ratio in COPD](http://www.ebi.ac.uk/gwas/search?query=Post bronchodilator FEV1/FVC ratio in COPD) (PMID: 26634245), [response to antipsychotic treatment in schizophrenia](http://www.ebi.ac.uk/gwas/search?query=Response to antipsychotic treatment in schizophrenia (working memory)) (PMID: 21107309), [partial epilepsies](http://www.ebi.ac.uk/gwas/search?query=Partial epilepsies) (PMID: 20522523) |
| *KRR1* | rs17115182 | | G/A (P43S) | [Polycystic ovary syndrome](http://www.ebi.ac.uk/gwas/search?query=Polycystic ovary syndrome) (PMID: 26416764), [blood Cu levels](http://www.ebi.ac.uk/gwas/search?query=Blood trace element (Cu levels)) (PMID: 23720494) |
| *PTCH2* | rs147284320 | | C/T (V503I) | None |
| *MUC17* | rs78010183 | | A/T (T1305S) | None |
| *SCN10A* | rs77804526 | | C/T (V1697I) | [Electrocardiographic traits](http://www.ebi.ac.uk/gwas/search?query=Electrocardiographic traits) (PMID: 25055868), [PR interval](http://www.ebi.ac.uk/gwas/search?query=PR interval) and [QRS duration](http://www.ebi.ac.uk/gwas/search?query=QRS duration) (PMID: 25035420), [QT interval](http://www.ebi.ac.uk/gwas/search?query=QT interval) (PMID: 24952745), [P wave duration](http://www.ebi.ac.uk/gwas/search?query=P wave duration) (PMID: 24850809) |
| *RFTN1* | rs180950245 | | C/G (N439K) | **Serum** [urate levels](http://www.ebi.ac.uk/gwas/search?query=Urate levels (BMI interaction)) (PMID: 25811787), optic nerve measurement (PMID: 20395239), [obesity](http://www.ebi.ac.uk/gwas/search?query=Obesity (extreme)) (PMID: 19553259) |
| *IGSF9B* | rs201459911 | | G/A (A1115V) | [Schizophrenia](http://www.ebi.ac.uk/gwas/search?query=Schizophrenia) (PMID: 26198764, PMID: 25056061), [longitudinal alcohol consumption](http://www.ebi.ac.uk/gwas/search?query=Longitudinal alcohol consumption) (PMID: 26081443), [obesity-related traits](http://www.ebi.ac.uk/gwas/search?query=Obesity-related traits) (PMID: 23251661) |
| *IQSEC3* | rs12822449 | | T/C (S283P) | [Diisocyanate-induced asthma](http://www.ebi.ac.uk/gwas/search?query=Diisocyanate-induced asthma) (PMID: 25918132), [response to angiotensin II receptor blocker therapy](http://www.ebi.ac.uk/gwas/search?query=Response to angiotensin II receptor blocker therapy) (PMID: 22566498) |
| *CCDC186* | rs79637542 | | C/T (A771T) | None |
| *PRAMEF12* | rs199576535 | | G/A (V341I) | None |
| 11p11.2 | rs1873059 | | G/A | [Chronic kidney disease](http://www.ebi.ac.uk/gwas/search?query=Chronic kidney disease) (PMID: 26831199), [thrombosis](http://www.ebi.ac.uk/gwas/search?query=Thrombosis) (PMID: 26908601), [Alzheimer disease and age of onset](http://www.ebi.ac.uk/gwas/search?query=Alzheimer disease and age of onset) (PMID: 26830138), [waist-to-hip ratio adjusted for body mass index](http://www.ebi.ac.uk/gwas/search?query=Waist-to-hip ratio adjusted for body mass index) (PMID: 26785701), [post-bronchodilator FEV1/FVC ratio in COPD](http://www.ebi.ac.uk/gwas/search?query=Post bronchodilator FEV1/FVC ratio in COPD) (PMID: 26634245) |
| *PTCHD3* | rs77473776 | | T/G (K186Q) | [Asthma](http://www.ebi.ac.uk/gwas/search?query=Asthma (childhood onset)) (PMID: 27142222), [epilepsy and lamotrigine-induced maculopapular eruptions](http://www.ebi.ac.uk/gwas/search?query=Epilepsy and lamotrigine-induced maculopapular eruptions) (PMID: 26220383), [fasting insulin-related traits (interaction with body mass index)](http://www.ebi.ac.uk/gwas/search?query=Fasting insulin-related traits (interaction with BMI)) (PMID: 22581228) |
| *L1TD1* | rs2886644 | | C/T (T613I) | None |
| Related to serum creatinine | |  | | |
| *CAT* | rs139421991 | | G/A (R320Q) | [Cataracts in type 2 diabetes](http://www.ebi.ac.uk/gwas/search?query=Cataracts in type 2 diabetes) (PMID: 23137000) |
| *EIF2AK4* | rs35602605 | | G/T (G1306C) | [Response to haloperidol in psychosis](http://www.ebi.ac.uk/gwas/search?query=Response to haloperidol in psychosis) (PMID: 24751813), [body mass index](http://www.ebi.ac.uk/gwas/search?query=Body mass index) (PMID: 24827717), [corneal curvature](http://www.ebi.ac.uk/gwas/search?query=Corneal curvature) (PMID: 22969067), [Ewing sarcoma](http://www.ebi.ac.uk/gwas/search?query=Ewing sarcoma) (PMID: 22327514) |
| *SP7* | rs188929035 | | G/A (A5V) | [Bone mineral density](http://www.ebi.ac.uk/gwas/search?query=Bone mineral density) (PMID: 22504420, PMID: 19801982, PMID: 19079262) |
| *CSMD2* | rs148658404 | | G/A (S3311F) | [Response to lithium treatment in bipolar disorder](http://www.ebi.ac.uk/gwas/search?query=Response to lithium treatment in bipolar disorder) (PMID: 26806518), [response to anti-TNF therapy in rheumatoid arthritis](http://www.ebi.ac.uk/gwas/search?query=Response to anti-TNF therapy in rheumatoid arthritis) (PMID: 26776603), [response to serotonin reuptake inhibitors in major depressive disorder](http://www.ebi.ac.uk/gwas/search?query=Response to serotonin reuptake inhibitors in major depressive disorder) (PMID: 25897834), [depressive symptoms](http://www.ebi.ac.uk/gwas/search?query=Depressive symptoms (SSRI exposure interaction)) (PMID: 25649181), [metabolite levels](http://www.ebi.ac.uk/gwas/search?query=Metabolite levels (HVA/MHPG ratio)) (PMID: 23319000) |
| *SASH1* | rs199980930 | | G/A | [Diabetic nephropathy](http://www.ebi.ac.uk/gwas/search?query=Type 2 diabetes nephropathy) (PMID: 21150874), [rotator cuff tears](http://www.ebi.ac.uk/gwas/search?query=Rotator cuff tears) (PMID: 26350878), [age-related hearing impairment](http://www.ebi.ac.uk/gwas/search?query=Age-related hearing impairment (interaction)) (PMID: 24939585), [smoking quantity](http://www.ebi.ac.uk/gwas/search?query=Smoking quantity) (PMID: 24665060), [thyroid hormone levels](http://www.ebi.ac.uk/gwas/search?query=Thyroid hormone levels) (PMID: 23408906) |
| *RNF123* | rs35620248 | | G/A (R387Q) | [Educational attainment](http://www.ebi.ac.uk/gwas/search?query=Educational attainment) (PMID: 25201988) |
| *ALG12* | rs3922872 | | T/C (I393V) | [Schizophrenia](http://www.ebi.ac.uk/gwas/search?query=Schizophrenia) (PMID: 26198764), [acne](http://www.ebi.ac.uk/gwas/search?query=Acne (severe)) (PMID: 24927181) |
| Related to CKD | |  | | |
| *VARS* | rs707926 | | G/A | None |
| *HDAC10* | rs112311672 | | G/A (T398M) | [Acne](http://www.ebi.ac.uk/gwas/search?query=Acne (severe)) (PMID: 24927181) |
| *ACAD11* | rs41272317 | | C/A | [Menarche](http://www.ebi.ac.uk/gwas/search?query=Menarche (age at onset)) (PMID: 25231870), [LDL-cholesterol](http://www.ebi.ac.uk/gwas/search?query=LDL cholesterol) and [HDL-cholesterol](http://www.ebi.ac.uk/gwas/search?query=HDL cholesterol) (PMID: 24097068) |

Data were obtained from GWAS Catalog (http://www.ebi.ac.uk/gwas) or GWAS Central (<http://www.gwascentral.org/browser)>. Kidney function–related phenotypes are shown in bold. PMID, PubMed ID.
